# Immuno-PET of epithelial ovarian cancer: harnessing the potential of CA125 for non-invasive imaging

**DOI:** 10.1186/s13550-014-0060-4

**Published:** 2014-11-12

**Authors:** Sai Kiran Sharma, Melinda Wuest, Monica Wang, Darryl Glubrecht, Bonnie Andrais, Suzanne E Lapi, Frank Wuest

**Affiliations:** Faculty of Pharmacy and Pharmaceutical Sciences, University of Alberta, 8613 - 114 Street, Edmonton, T6G 2H1 AB Canada; Department of Oncology, University of Alberta, 11560 University Avenue, Edmonton, T6G 1Z2 AB Canada; Mallinckrodt Institute of Radiology, Washington University, 510 South Kingshighway Boulevard, St. Louis, 63110 MO USA

**Keywords:** Antibody, CA125, Copper-64, Positron emission tomography, Epithelial ovarian cancer

## Abstract

**Background:**

Epithelial ovarian cancer (EOC) is characterized by the overexpression of cancer antigen 125 (CA125), a mucinous glycoprotein that serves as a tumor biomarker. Early diagnosis of EOC is plagued by its asymptomatic nature of progression and the limitations of currently used immunoassay techniques that detect CA125 as a shed antigen in serum samples. Presently, there is no technique available for the *in vivo* evaluation of CA125 expression in malignant tissues. Moreover, there could be an unexplored pathophysiological time window for the detection of CA125 in EOC, during which it is expressed on tumor cells prior to being shed into the bloodstream. A method for the *in vivo* evaluation of CA125 expression on ovarian neoplasms earlier along disease progression and/or recurrence can potentially contribute to better disease management. To this end, the present work utilizes an anti-CA125 monoclonal antibody (MAb) and a single-chain variable fragment (scFv) labeled with the positron-emitting radionuclide ^64^Cu for preclinical molecular imaging of CA125 expression *in vivo*.

**Methods:**

Anti-CA125 MAb and scFv were prepared and functionally characterized for target binding prior to being tested as radiotracers in a preclinical setting.

**Results:**

Immunoblotting, immunofluorescence, and flow cytometry revealed specific binding of CA125-targeting vectors to NIH:OVCAR-3 cells and no binding to antigen-negative SKOV3 cells. ^64^Cu-labeled anti-CA125 MAb and scFv were obtained in specific activities of 296 and 122 MBq/mg, respectively. Both radioimmunoconjugate vectors demonstrated highly selective binding to NIH:OVCAR-3 cells and virtually no binding to SKOV3 cells. *In vivo* radiopharmacological evaluation using xenograft mouse models injected with ^64^Cu-labeled anti-CA125 MAb provided a standardized uptake value (SUV) of 5.76 (29.70 %ID/g) in OVCAR3 tumors 24 h post-injection (p.i.) versus 1.80 (5.91 %ID/g) in SKOV3 tumors. ^64^Cu-labeled anti-CA125 scFv provided an SUV of 0.64 (3.21 %ID/g) in OVCAR3 tumors 24 h p.i. versus 0.25 (1.49 %ID/g) in SKOV3 tumors. Results from small-animal PET imaging were confirmed by *ex vivo* autoradiography and immunohistochemistry.

**Conclusions:**

Radiolabeling of anti-CA125 MAb and scFv with ^64^Cu did not compromise their immunoreactivity. Both radioimmunoconjugates presented specific tumor uptake and expected biological clearance profiles. This renders them as potential immuno-PET probes for targeted *in vivo* molecular imaging of CA125 in EOC.

**Electronic supplementary material:**

The online version of this article (doi:10.1186/s13550-014-0060-4) contains supplementary material, which is available to authorized users.

## Background

Despite its relative rarity in the general population and the availability of standard treatment through surgical intervention and platinum chemotherapy, epithelial ovarian cancer (EOC) continues to be the most insidious and lethal gynecologic malignancy in the western world. The Surveillance Epidemiology and End Results (SEER) program has estimated 21,980 new cases of ovarian cancer to occur in 2014, with 14,270 deaths resulting from this disease in the United States alone [1]. A combination of the asymptomatic nature of this malignancy and the high incidence of recurrence results in a low 5-year survival rate of 44.6% [1].

Cancer antigen 125 (CA125) is a high molecular weight mucinous glycoprotein overexpressed on the membrane of EOC cells [2]-[4]. It has clinical relevance as a standard-of-care serum biomarker for ovarian cancer surveillance despite expression in some non-gynecologic malignancies and benign conditions such as pregnancy, menstruation, endometriosis, liver disease, and congestive heart failure [3],[4]. Post-treatment elevation of serum CA125 level in EOC patients serves as an indicator of progressive disease and finds clinical application in the management of patients with documented evidence of ovarian cancer [3]-[7]. However, a normal CA125 serum titer does not necessarily imply an absence of the disease. This is exemplified by the fact that an estimated 50% of patients with normal levels of CA125 post-chemotherapy have small volumes of active disease at second-look surgery, and a large portion of this population succumbs to recurrence from the disease [5],[6]. Presently, serial CA125 levels in patients are evaluated using immunoassays.

However, despite their indicative utility, such assays can be far from delivering a true representation of the *in vivo* pathophysiological pattern and may be limited in their capabilities for early detection of ovarian neoplasms.

On a time scale of events for the diagnosis of recurrent ovarian cancer, it is known that radiological detection of the disease precedes the presentation of clinically diagnosable symptoms by 2 to 3 months. Furthermore, there is a median lag of 2 months between the elevation of CA125 alone and the radiological detection of this disease [5],[6]. Among contemporary imaging modalities, [^18^F] FDG-PET has shown the highest sensitivity and accuracy in detecting recurrent ovarian cancer, while simultaneous investigations with CT, MRI, and ultrasound which primarily detect morphological features and anatomical changes that can be distorted on account of primary cyto-reductive surgery in patients yielded negative or equivocal results [8]-[11].

Considering these facts, we hypothesized that a non-invasive approach using targeted immuno-PET to directly image the *in vivo* overexpression of CA125 on epithelial ovarian neoplasms may detect the malignancy at an earlier time, while providing a more accurate *in vivo* evaluation of tumor load and residual disease. Further, this approach could gain relevance when used in a pathophysiological time window wherein CA125 expression is limited to the surface of ovarian neoplasms prior to its shedding into the bloodstream. Our hypothesis is strengthened further by a mathematical model proposed by Hori and Gambhir [12] whereby an ovarian tumor may grow for 10.6 years before attaining a size of 10.52 mm^3^ before it starts to shed just enough CA125 antigen (1.5 U/mL) detectable by immunoassay methods used in the clinic.

Owing to its ability for targeting CA125 in ovarian cancer, MAb-B43.13 has been employed as an immunotherapeutic agent in the treatment of EOC [13]-[15]. More recently, immuno-PET has emerged as a strategy that unifies the specificity of antibody-based targeting with the sensitivity for detection imparted by positron-emitting radionuclides in PET in order to render a superlative diagnostic potential [16].

Findings from previous research with MAb-B43.13 and more recent reports for antibody-based radiotracers targeting biomarkers such as PSMA [17] and CA19-9 [18], which have similar pathophysiological nature in prostate and pancreatic cancers, respectively, motivated us to develop an immuno-PET strategy for the *in vivo* imaging of EOC. Additionally, we developed an antibody fragment of MAb-B43.13, in order to produce a radiotracer that could retain antigen-binding properties like the full-length antibody but achieve faster *in vivo* clearance and better tumor penetration as a function of its smaller molecular size, which may ultimately yield high-contrast *in vivo* PET images at earlier time points post-injection. To this end, MAb-B43.13 and its derivative single-chain variable fragment (scFv-B43.13) were labeled with positron emitter ^64^Cu (*t*_1/2_ 12.7 h) and analyzed *in vitro* and *in vivo* using CA125 overexpressing NIH:OVCAR-3 cells and CA125-negative SKOV3 cells in preclinical EOC xenograft mouse models.

## Methods

### Expression and purification of anti-CA125 MAb and scFv

The murine monoclonal IgG1 antibody targeting human CA125 was produced from hybridoma B43.13 [19] that was kindly provided by Quest Pharma Tech Inc., Edmonton, Canada. The hybridoma cell culture supernatant was used to purify the MAb-B43.13 by protein G affinity (P-7700, Sigma-Aldrich, St. Louis, MO, USA) on a BioLogic DuoFlow™ chromatography system (760-0135, Bio-Rad Laboratories, Inc., Hercules, CA, USA). MAb-B43.13-derived anti-CA125 scFv was produced with modifications to previously described constructs [20],[21]. Briefly, the DNA sequence encoding scFv-B43.13 was cloned into a pET22b + vector with a modified inter-chain linker, and the protein was expressed in *Escherichia coli* Rosetta 2 DE3 (Novagen, 71400-3, Merck KGaA, Darmstadt, Germany). The C-terminal hexa-histidine-tagged scFv was purified from the soluble fraction of recombinant cell lysates by immobilized metal affinity chromatography using a TALON® Superflow resin (635507, Clontech Laboratories, Inc., Mountain View, CA, USA).

### Cell lines and culture conditions

Ovarian cancer cell lines NIH:OVCAR-3 (ATCC® HTB-161™, ATCC, Manassas, VA, USA) and SKOV3 (ATCC® HTB-77™) were used for *in vitro* and *in vivo* studies. Cells were cultured in DMEM-F12 medium supplemented with 10% *v*/*v* fetal bovine serum (FBS), 50 IU/mL penicillin, and 50 μg/mL streptomycin (Gibco®, Life Technologies, Carlsbad, CA, USA). NIH:OVCAR-3 cells were additionally supplemented with 7 μg/mL recombinant human insulin (91077C, SAFC Biosciences, Inc., Lenexa, KS, USA). Cells were cultured using sterile techniques and grown in a 37°C incubator providing humidified atmosphere of 5% CO_2_ in air.

### Characterization of CA125-targeting vectors

#### Western blotting

NIH:OVCAR-3 and SKOV3 cells (7.5 × 10^6^) were lysed with CelLytic™ M (C2978, Sigma-Aldrich). The cell lysates were electrophoresed on a 4% to 15% Mini-PROTEAN® TGX™ precast gel (456-1085, Bio-Rad) and transferred to a Trans-Blot nitrocellulose membrane (162-0115, Bio-Rad). The membranes were probed separately to evaluate MAb versus scFv binding to the target antigen in cell lysates. The blots were blocked for 45 min with 5% non-fat dry milk (Carnation) in PBS having 0.1% Tween-20 (PBST). Anti-CA125 MAb (3 mg/mL), mouse anti-β actin IgG (A1978, Sigma-Aldrich), and anti-CA125 scFv (3 mg/mL) were used as primary antibodies to probe the blots at 1:5,000 dilutions for 1 h at room temperature. Goat anti-mouse HRP conjugate (A4416, Sigma-Aldrich) was used as secondary antibody to probe the blot against anti-CA125 MAb and mouse anti-β actin IgG at 1:5,000 dilution for 1 h at room temperature. 6xHis MAb-HRP conjugate (631210, Clontech) was used as secondary antibody at 1:5,000 dilution to probe against anti-CA125 scFv for 1 h at room temperature. The blots were washed with PBST and developed on Amersham Hyperfilm ECL (28906839, GE Healthcare, Little Chalfont, UK) using Amersham ECL Plus Western Blotting Detection Reagents (RPN2132, GE Healthcare).

#### Fluorescent labeling of anti-CA125 MAb/scFv

One milligram each of anti-CA125 MAb and scFv at concentrations of 2 mg/mL were fluorescently labeled using the Pierce® fluorescein isothiocyanate (FITC) antibody labeling kit (53027, Thermo Scientific, Waltham, MA, USA) according to the manufacturer's instructions.

#### Flow cytometry

NIH:OVCAR-3 cells (1.5 × 10^6^) were harvested by trypsinization, rinsed twice with fluorescence-activated cell sorting (FACS) buffer (PBS with 0.5% heat-inactivated FBS, 2 mM EDTA, 0.05% sodium azide), and re-suspended by gentle tapping in an approximately 100 μl FACS buffer. Ten micrograms of anti-CA125 MAb or scFv was incubated with the cell suspension for 30 min at room temperature. Cells were rinsed twice in FACS buffer and incubated for 30 min with 4 μg of Alexa Fluor® 488 goat anti-mouse antibody (A-11001, Life Technologies) for the MAb samples and 4 μg of Penta • His Alexa Fluor 488 conjugate (35310, Qiagen, Venlo, the Netherlands) for the scFv samples. Cells were rinsed twice with FACS buffer and analyzed by flow cytometry on a BD FACS Calibur (BD Biosciences, San Jose, CA, USA). A dengue virus targeting IgG1-12A1 and a hexa-histidine-tagged anti-RANK receptor binding scFv were used as isotype controls for the experiments. Negative controls included unstained NIH:OVCAR-3 cells and cells incubated with Alexa Fluor 488 conjugated antibodies alone.

#### Immunofluorescence

NIH:OVCAR-3 and SKOV3 cells were plated onto glass coverslips in 35-mm tissue culture dishes (100,000 cells/2 mL medium/dish) and incubated at 37°C for 48 h. The cells were rinsed with PBS and fixed in methanol for 30 min at -20°C. The fixed cells were incubated in 5% non-fat dry milk (Carnation) in PBS for 30 min and immunostained separately for 1 h with FITC-labeled versus unlabeled anti-CA125 MAb and scFv. The unlabeled MAb and scFv samples were indirectly stained with corresponding Alexa Fluor 488-labeled secondary antibodies as used for flow cytometry experiments.

Anti-CA125 MAb and scFv (2 mg/mL) were used at 1:250 dilution followed by 1:500 dilutions of secondary antibodies (2 mg/mL) in PBS containing 5% non-fat dry milk. Appropriate blank and control samples were included in the experiments. Antibody incubations were followed by three rinses with PBST for 5 min each. Coverslips were mounted on microscopy slides (Fisherbrand, 12-550-003, Thermo Fisher Scientific) using Mowiol® mounting medium (Calbiochem, 475904, Millipore Co., Billerica, MA, USA) supplemented with DAPI (50 μg/mL). Immunofluorescence was observed through a Zeiss Plan Apochromat 40X/1.3 Oil DIC M27 lens on a confocal laser scanning microscope (Zeiss LSM 710, Carl Zeiss AG, Oberkochen, Germany), and images were analyzed using Zen 2011 software. Separately, 30 μg of FITC-labeled anti-CA125 MAb was added to NIH:OVCAR-3 cells grown on coverslips and allowed to incubate under standard cell culture conditions over a period of 48 h to study uptake in live cells. Coverslips were washed and mounted on glass slides for analysis by confocal microscopy at 0.5-, 1-, 4-, 12-, 24-, and 48-h time points.

#### Surface plasmon resonance

Kinetic constants for association (*k*_a_) and dissociation (*k*_d_) and affinity constant *K*_D_ for anti-CA125 MAb-B43.13 and scFv-B43.13 were determined by surface plasmon resonance on a Biacore 3000 instrument (GE Life Sciences, Piscataway, NJ, USA). Upon activation of a CM5 sensor chip with a 1:1 mixture of *N*-hydroxysuccinimide/*N*-ethyl-*N*'-(3-dimethylaminopropyl)-carbodiimide hydrochloride, amine coupling of antigen-grade human ovarian cancer native CA125 protein (MBS318371, MyBioSource, Inc., San Diego, CA, USA) dissolved in 10 mM sodium acetate buffer pH 5.0 was performed to couple approximately 1,200 relative units. The remaining reactive groups in the control and test lanes were inactivated using ethanolamine from the amine coupling kit (GE Life Sciences). The binding of antibody vectors to CA125 immobilized on the CM5 chip was assessed in duplicates across concentration ranges between 18.7 nM and 9.33 μM for MAb-B43.13 and 19.6 nM and 19.6 μM for the scFv-B43.13, respectively. Each sample of MAb/scFv in the aforementioned concentrations in binding buffer (10 mM HEPES, pH 7.0) was injected for 3 min at a flow rate of 30 μL/min to allow binding with the antigen. The binding buffer was allowed to flow over the sensor chip for 15 min at a rate of 30 μL/min to allow for the dissociation of bound MAb/scFv from the antigen on the chip. Next, the regeneration buffer (10 mM HEPES pH 7.0 supplemented with 800 mM KCl) was passed over the chip surface to achieve dissociation of any remaining bound analyte, followed by 2 min for stabilization before the next injection. BIAcore control software 3.2 was used to analyze the data, and the best 1:1 Langmuir binding fit was used to derive kinetic constants.

### Preparation of CA125-targeting radioimmunoconjugates

#### General

All glassware was rinsed with ultra-pure HCl (Fisherbrand, A508-P500, Thermo Fisher Scientific). Trace metal basis ultra-pure chemicals for buffer preparations were purchased from Sigma-Aldrich. All buffer solutions were treated with biotechnology-grade Chelex 100 (143-2832, Bio-Rad).

#### NOTA functionalization

*p*-SCN-Bn-NOTA [*S*-2-(4-isothiocyanatobenzyl)-1,4,7-triazacyclononane-1,4,7-triacetic acid] (B-605, Macrocyclics, Dallas, TX, USA) was conjugated to anti-CA125 MAb and scFv to serve as a bifunctional chelator for ^64^Cu radiolabeling. Briefly, a 6 M excess of p-SCN-Bn-NOTA in DMSO was added to anti-CA125 MAb/scFv in 0.1 M sodium bicarbonate buffer pH 9.0 and allowed to react for 1 h at 37 C. NOTA-functionalized anti-CA125 MAb/scFv was purified from excess unconjugated bifunctional chelator while simultaneously achieving buffer exchange into 0.25 M sodium acetate pH 5.5 by using an Econo-Pac 10DG desalting column (732-2010, Bio-Rad). Protein quantification of the column-eluted fractions was performed using A_280_ measurements on nanodrop 2000 (Thermo Scientific) and bicinchoninic assay (Pierce BCA Protein Assay kit, 23227) using a Synergy H1 multimode microplate reader for A_562_ absorbance read-out. The number of bifunctional chelates conjugated per MAb and scFv was determined by matrix-assisted laser desorption-ionization time-of-flight (MALDI-TOF) MS analysis and according to the method described by Cooper et al. [22]. The purified immunoconjugates were used in radiolabeling experiments.

#### Cu labeling of NOTA-functionalized MAb/scFv

^64^Cu was produced via a ^64^Ni(p, n)^64^Cu nuclear reaction on a CS-15 biomedical cyclotron at Washington University, St. Louis, USA as previously described [23] and supplied as high specific activity ^64^CuCl_2_ in 0.1 N HCl. Ammonium acetate (0.1 M; pH 5.5) was added to ^64^CuCl_2_ to form ^64^Cu-acetate ^64^Cu (OAc)_2_ solution. To 100 μg of NOTA-functionalized MAb/scFv, 85 MBq of ^64^Cu (OAc)_2_ was added and allowed to react on a thermomixer at 30°C, 550 rpm for 1 h. EDTA (1 mM) was added to quench the reaction over 10 min. ^64^Cu-labeled MAb/scFv radioimmunoconjugates were purified on an Econo-Pac 10DG desalting column pre-equilibrated with 0.25 M sodium acetate, pH 5.5 used as the eluant. Elution fractions (350 μL) were collected from the column, and the radioactivity was measured using an Atomlab 400 dose calibrator (Biodex, Shirley, NY, USA). Fifteen microliters of each elution fraction was electrophoresed on a 12% SDS-PAGE gel under non-reducing conditions and evaluated by autoradiography on a BAS-5000 phosphorimager (Fujifilm, Tokyo, Japan). Radiochemical yields and purity were determined by instant thin layer chromatography on ITLC-SG (SGI001, Varian, Inc., Palo Alto, CA, USA), using 10 mM EDTA pH 5.5 as the eluant. Fractions containing high specific activity radioimmunoconjugates were used for *in vitro* and *in vivo* radiopharmacological experiments. Monoclonal IgG1 antibody 12A1 was modified into an immunoconjugate and radiolabeled with ^64^Cu as described above to serve as a non-specific isotype control for *in vivo* experiments.

### Functional characterization of CA125-targeting radioimmunoconjugates

#### Cell uptake studies

NIH:OVCAR-3 and SKOV3 cells were seeded to obtain 250,000 cells per well in 12-well tissue culture plates. Prior to the uptake experiment, the growth medium was removed and cells were rinsed twice with PBS and incubated in Krebs buffer at 37 C for 1 h. The radioimmunoconjugate (100 KBq) was added to each well except those assigned to measure background activity alone. Cell uptake was terminated at 5, 10, 15, 30, 60, 90, and 120 min by adding ice-cold Krebs buffer and rinsing the wells twice to wash away any unbound radioimmunoconjugates prior to lysing the cells with RIPA buffer.

The cell lysates were transferred to scintillation vials and measured for radioactivity using a γ-counter (Wizard^2^® 2480 Automatic Gamma Counter, PerkinElmer, Ontario, Canada). Protein levels were quantified using a Pierce™ BCA protein assay kit according to the manufacturer's recommendations. Cell uptake levels were normalized to percentage of the total amount of radioactivity per milligram of protein (% radioactivity/mg protein) and plotted as a function of time. All experiments were performed in triplicates.

Immunoreactivity of the radioimmunoconjugates was assessed by cell binding assays according to the method of Lindmo et al. [24].

#### *In vivo* experiments

##### Xenograft models

All animal experiments were carried out according to guidelines of the Canadian Council on Animal Care (CCAC) and approved by the local animal care committee of the Cross Cancer Institute. Six-week-old BALB/c nude female mice were obtained from Charles River labs (Quebec, Canada). The animals were housed in ventilated cages and provided food and water *ad libitum*. NIH:OVCAR-3 tumors were induced on the left shoulder by two subcutaneous injections of 15 × 10^6^ and 10 × 10^6^ cells in a 300 μL suspension of 1:1 mixture of PBS and Matrigel (354234, BD Biosciences). The second injection of cells was administered at the same site within 7 to 10 days. NIH:OVCAR-3 tumors grew for 6 to 8 weeks before reaching suitable tumor sizes of 150 to 200 mm^3^.

SKOV3 tumors were induced on the left shoulder by a single subcutaneous injection of 5 × 10^6^ cells in PBS and were grown for about 2 to 3 weeks before achieving similar tumor sizes.

#### Animal imaging

Small-animal PET experiments were performed on a MicroPET® R4 or INVEON PET scanner (Siemens Preclinical Solutions, Knoxville, TN, USA). Mice were anesthetized by inhalation of isoflurane in 40% oxygen/60% nitrogen, 1 L/min, while maintaining body temperature at 37°C throughout the experiment; 6 to 10 MBq of high specific activity radioimmunoconjugate (approximately 30 to 40 μg) in 150 to 200 μL of 0.25 M sodium acetate (pH 5.5) was administered intravenously via a tail vein catheter. For blocking experiments, 1 mg of unmodified anti-CA125 MAb was administered intraperitoneally 24 h prior to injection of the ^64^Cu-labeled radioimmunoconjugates. In separate animals, 8 to 10 MBq of ^64^Cu-labeled 12A1 IgG1 was administered via tail vein injection to evaluate non-specific uptake. Whole-body PET data was acquired by performing static scans for each animal at 24 h post-injection (p.i.) and 48 h p.i. Data acquisition continued for 60 min in 3D list mode. The image files were reconstructed using the maximum a posteriori (MAP) algorithm. The image files were further processed using the ROVER v 2.0.51 software (ABX GmbH, Radeberg, Germany). Masks for defining 3D regions of interest (ROI) were set, and the ROIs were defined by thresholding. Standardized uptake values [SUV = (activity/mL tissue)/(injected activity/body weight), mL g^-1^] were calculated for each ROI. Additionally, radioactivity uptake was also analyzed as percentage of injected dose per gram tissue (%ID/g). Data are expressed as means ± SEM from *n* investigated animals.

#### *Ex vivo* analyses

An NIH:OVCAR-3 tumor-bearing mouse was injected with 4.55 MBq of ^64^Cu-labeled anti-CA125 MAb via the tail vein. At 24 h p.i., the animal was euthanized by cervical dislocation. The tumor was submerged into OCT medium and flash frozen using a bath of dry ice-cooled methanol. Seven-micrometer slices were cut on a Leica CM 1850 cryostat (Leica Microsystems, Singapore).

#### Autoradiography

Tissue sections were placed into a BAS-Cassette (2325, Fujifilm) and exposed to a phosphor imaging plate (BAS-MS 2025, Fujifilm) for 15 h at room temperature. The images were developed on a BAS-5000 reader (Fuji Photo Film Co., Ltd.) and analyzed with Adaptive Image Deconvolution Algorithm (AIDA) Image Analyzer v.450 software.

#### Immunofluorescence

Upon thawing, tissue sections were fixed with formalin for 30 min. The fixed sections were blocked overnight at 4°C using 0.12 mg/mL unconjugated goat anti-mouse Fab fragment (115-003-007, Jackson Immunoresearch, West Grove, PA, USA) in 0.5% fish skin gelatin (G7765, Sigma-Aldrich) pH 7.4, supplemented with 0.1% Triton X-100. The sections were rinsed 3× with Tris-buffered saline having 0.5% Tween 20 (TBST) for 5 min each. MAb-B43.13 was used as primary antibody at a dilution of 1:6,000 in Dako antibody diluent (S0809, Dako, Glostrup, Denmark) and allowed to incubate overnight at 4 C. The sections were rinsed 3× with TBST and incubated with Alexa Fluor® 488 goat anti-mouse antibody (A-11001, Life Technologies) used as secondary antibody at 1:400 dilution in Dako antibody diluent for 2 h at room temperature. After three washes with TBST, the sections were rinsed with water and counterstained using Hoechst H33342 (2 μg/mL) for 5 min. The sections were rinsed and mounted under a coverslip using FluorSave (345789, Calbiochem). The slides were analyzed with a Zeiss Plan Apochromat 10X/0.45 na lens on a confocal laser scanning microscope (Zeiss LSM 710) using Z-stack for image acquisition. The images were registered using Zen 2011 software (Zeiss) and further processed with Adobe Photoshop CS6.

#### Immunohistochemistry

To analyze the samples for immunohistochemistry, the same procedures for treatment, fixation, blocking, and probing of tissue with primary antibody were followed. DakoEnVision™ + HRP-conjugated anti-mouse antibody was used as a secondary antibody, and no counterstain was employed. The sections were observed with a Zeiss Fluar 2.5X/0.12 na lens on an Axioskop 2 Plus microscope. Images were processed and analyzed on AxioVision 4.7 (Zeiss) and further processed with Adobe Photoshop CS6.

### Statistical analysis

All data are expressed as means ± SEM. Graphs were constructed using GraphPad Prism 4.0 (GraphPad Software). Where applicable, statistical differences were tested by unpaired Student's *t* test and were considered significant for *p* <0.05.

## Results

### Isolation, characterization, and modification of CA125-targeting vectors

**Figure 1:**
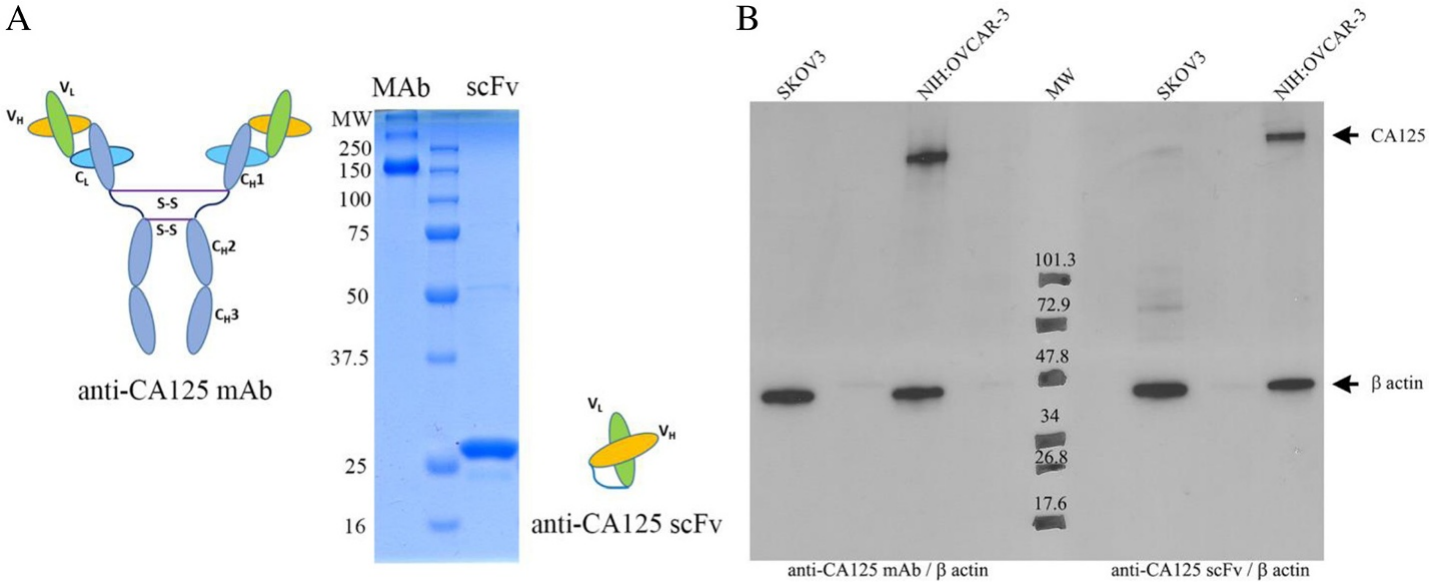
**Representative images and Western blots. (A)** Representative images of purified anti-CA125 MAb (148 KDa) and scFv (28 KDa) upon SDS-PAGE under non-reducing conditions. **(B)** Western blots of SKOV3 (CA125^-^) and NIH:OVCAR-3 (CA125^+^) cells with anti-CA125 MAb, anti-β actin antibody (left side of molecular weight marker); anti-CA125 scFv, and anti-β actin antibody (right side of molecular weight marker).

**Figure 2:**
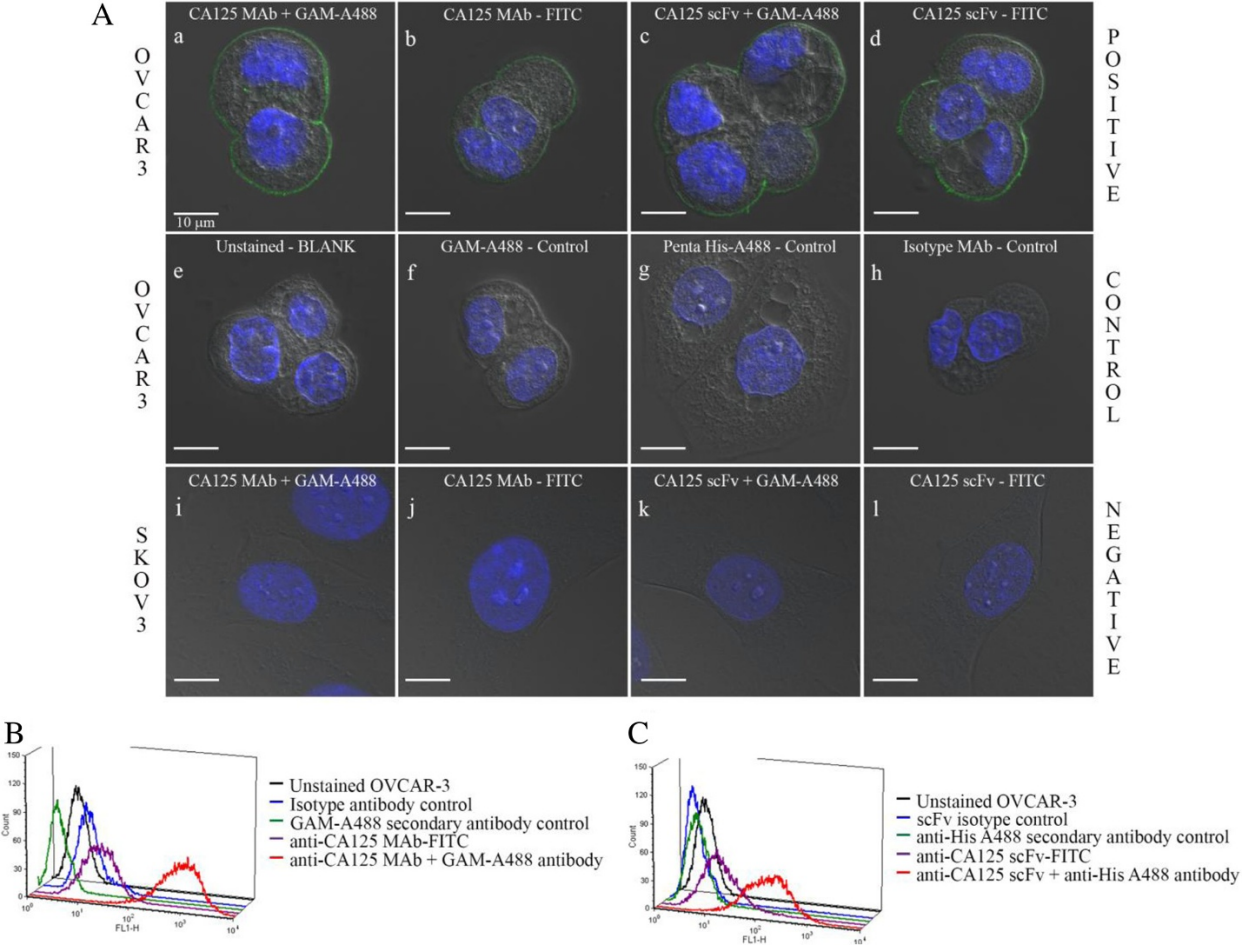
**Confocal microscopy and flow cytometry. (A)** Confocal microscopy images from immunofluorescence studies. (a-b) Immunostaining of NIH:OVCAR-3 cells with unlabeled versus FITC-labeled anti-CA125 MAb. Goat anti-mouse Alexa Fluor 488 IgG (GAM-A488) was used as secondary antibody. (c-d) Immunostaining of NIH:OVCAR-3 cells with unlabeled versus FITC-labeled anti-CA125 scFv. Penta-His Alexa Fluor 488 antibody was used as secondary antibody. (e) Unstained NIH:OVCAR-3 cells - blank. (f) NIH:OVCAR-3 cells stained with GAM-A488 antibody - negative control. (g) NIH:OVCAR-3 cells stained with Penta-His Alexa Fluor 488 antibody - negative control. (h) Immunostaining of NIH:OVCAR-3 cells with MAb 12A1 and GAM-A488 antibody - isotype control. (i-j) Immunostaining of SKOV3 cells with unlabeled versus FITC-labeled anti-CA125 MAb. (k-l) Immunostaining of SKOV3 cells with unlabeled versus FITC-labeled anti-CA125 scFv. Scale bar represents 10 μm. Flow cytometry analysis of **(B)** anti-CA125 MAb and **(C)** anti-CA125 scFv binding to NIH:OVCAR-3 cells.

CA125-targeting vectors were purified in yields of 6 mg/L for MAb-B43.13 and 0.7 mg/L for the scFv (Figure 1A). Analysis by Western blot (Figure 1B) and immunofluorescence staining (Figure 2A) ascertained the following: a) expression of CA125 in NIH:OVCAR-3 cells, b) absence of CA125 expression in SKOV3 cells, and c) binding of MAb and scFv to target antigen CA125. Isothiocyanate-based labeling of anti-CA125 MAb and scFv with fluorescein yielded 2 mol of FITC per mole of MAb and scFv. SPR analysis calculated an affinity constant of 2.56 × 10^-9^ M for MAb-B43.13 and 1.11 × 10^-8^ M for the scFv, with comparable dissociation constants in the range of 10^-4^s^-1^ (Additional file 1: Table S1).

Immunofluorescence staining and flow cytometry analysis with the FITC-labeled MAb and scFv demonstrated preserved immunoreactivity by binding to CA125 antigen expressed on the membrane of NIH:OVCAR-3 cells, and no binding was seen with SKOV3 cells (Figure 2A, B, C).

**Figure 3:**
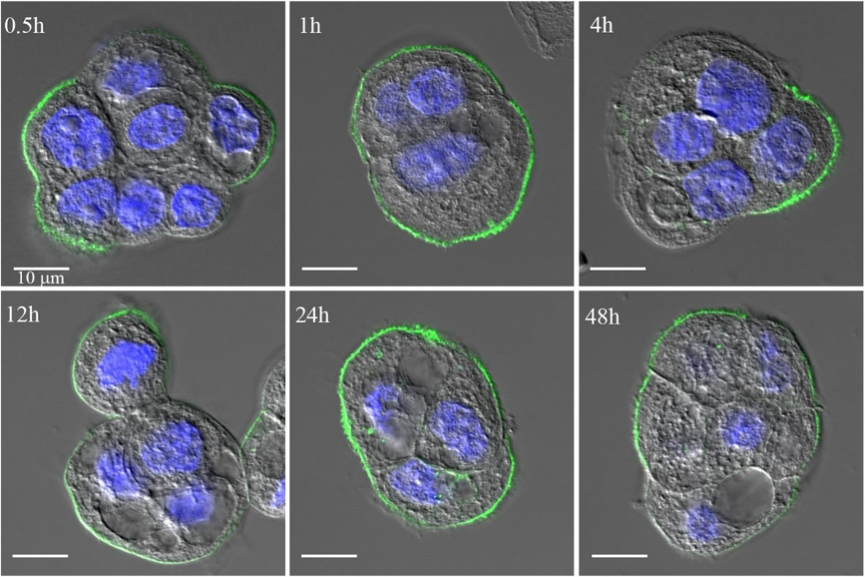
**Confocal microscopy images of NIH:OVCAR-3 cells directly immunostained with FITC-labeled anti-CA125 MAb over 48 h.**

FITC-labeled anti-CA125 MAb remained membrane bound over a period of 48 h with minimal cellular internalization observed upon incubation with live NIH:OVCAR-3 cells in culture (Figure 3).

### Development and functional assessment of CA125-targeting radioimmunoconjugates

**Figure 4:**
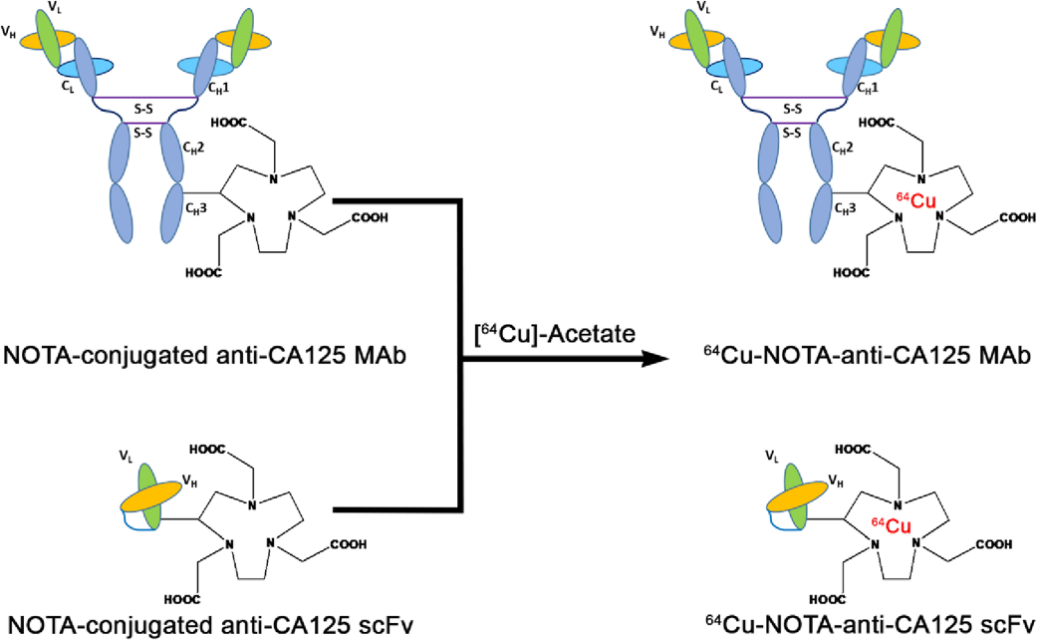
**Schematic representation of** ^**64**^ **Cu-labeling of NOTA conjugated anti-CA125 MAb and scFv.**

MALDI-TOF analyses and isotopic dilution assays revealed an average of 2.4 NOTA molecules conjugated per MAb and 1.9 NOTA molecules conjugated per scFv (Additional file 1: Figures S2 and S3). ^64^Cu-labeling and purification of radioimmunoconjugates provided isolated radiochemical yields of 65% ±8% (*n* = 14) with specific activity of 296 ± 37 MBq/mg for anti-CA125 MAb and 56% ±14% (*n* = 9) with specific activity of 122 ± 44 MBq/mg for anti-CA125 scFv (Figure 4).

**Figure 5:**
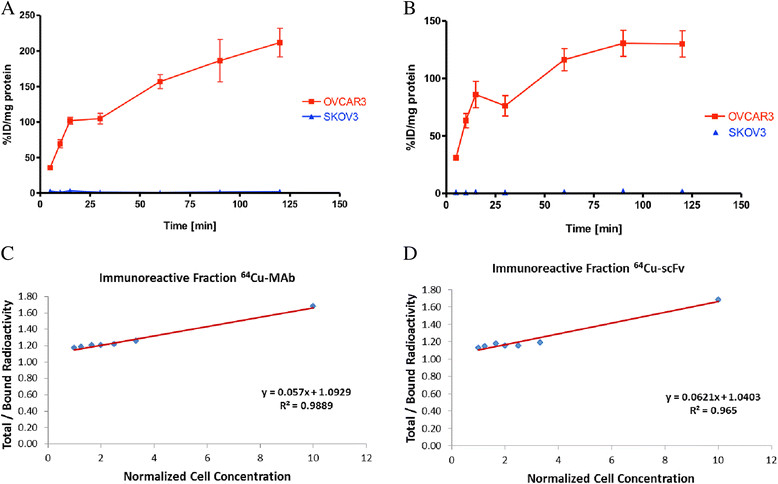
**Cell uptake and double inverse plots.** Graphs for cell uptake of **(A)**^64^Cu-labeled anti-CA125 MAb and **(B)**^64^Cu-labeled anti-CA125 scFv in NIH:OVCAR-3 and SKOV3 cells. Representative double inverse plots from Lindmo assays performed with **(C)**^64^Cu-labeled anti-CA125 MAb and **(D)**^64^Cu-labeled anti-CA125 scFv in NIH:OVCAR-3 cells.

Radiochemical purity of the isolated radioimmunoconjugates was greater than 99% as analyzed by ITLC (Additional file 1: Figure S4) and phosphor images of electrophoresed fractions (Additional file 1: Figure S5). Purified radioimmunoconjugates were >95% stable in human AB-type serum over 48 h (Additional file 1: Figures S6 and S7) and demonstrated highly specific binding to CA125 expressed on NIH:OVCAR-3 cells with virtually no binding to SKOV3 cells as evaluated by cell uptake studies (Figure 5A, B). Immunoreactivity of ^64^Cu-labeled anti-CA125 MAb and scFv preparations was found to be >90% as obtained from the inverse of the intercept of plots seen in Figure 5C, D, respectively.

### *In vivo* experiments and radiopharmacological evaluation

**Figure 6:**
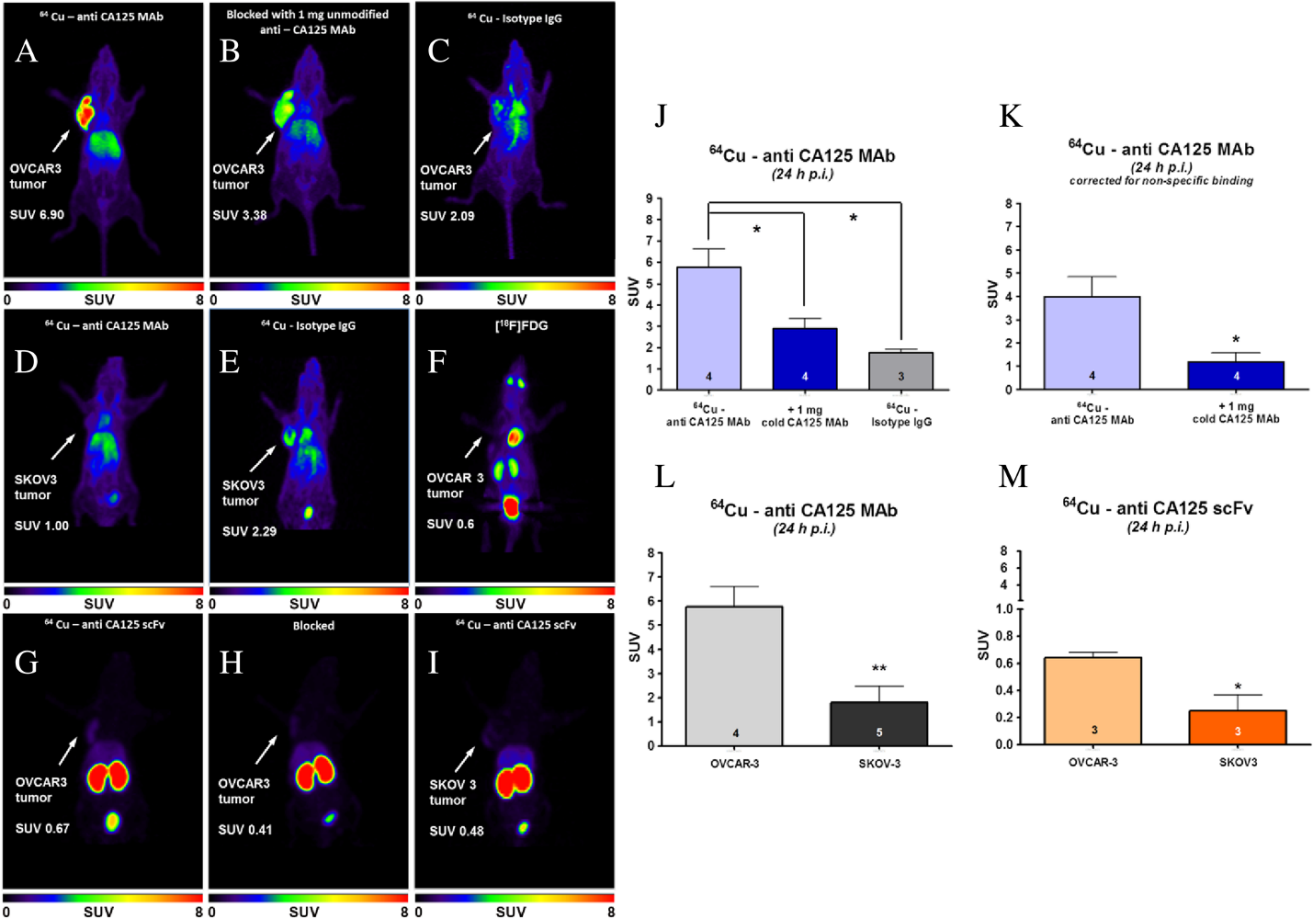
**PET images and diagrams. (A-E**, **G-I)** Representative 24-h post-injection small-animal-PET images of xenograft mice injected with radioimmunoconjugates. **(F)** 1-h p.i. PET image of NIH:OVCAR-3 xenograft mouse injected with ^18^ F-FDG. Color intensity scale bars represent standardized uptake value (SUV) of radiotracer in animals. Diagrams on the right show **(J)** SUV in tumors of experimental and control animals using ^64^Cu-labeled anti-CA125 MAb, **(K)** SUV in NIH:OVCAR-3 tumors after correction for non-specific uptake, **(L)** tumor SUV obtained from injection of ^64^Cu-labeled anti-CA125 MAb in NIH:OVCAR-3 versus SKOV3 xenograft mice, and **(M)** tumor SUV obtained from injection of ^64^Cu-labeled anti-CA125 scFv in NIH:OVCAR-3 versus SKOV3 xenograft mice. Numbers of animals (*n*) are indicated at the bottom of each bar.

PET imaging studies performed with ^64^Cu-labeled anti-CA125 MAb and scFv in NIH:OVCAR-3 and SKOV-3 tumor-bearing mice as well as experiments with ^64^Cu-labeled isotype IgG and [^18^F] FDG are summarized in Figure 6.

In NIH:OVCAR-3 tumors, ^64^Cu-labeled anti-CA125 MAb reached a maximum SUVmean of 5.76 ± 0.85 (29.70 ± 3.34 %ID/g) at 24 h p.i. rising to 7.06 ± 0.67 (41.66 ± 4.38 %ID/g) at 48 h p.i. (*n* = 4). NIH:OVCAR-3 xenograft mice pre-dosed with unlabeled MAb revealed a SUVmean of 2.90 ± 0.45 (13.10 ± 2.07 %ID/g) at 24 h p.i. (*n* = 4, *p* <0.05) and 3.96 ± 0.76 (14.47 ± 3.01 %ID/g) at 48 h p.i. (*n* = 4, *p* <0.05). Non-specific tumor uptake using ^64^Cu-labeled isotype IgG1 was found to have a SUVmean of 1.77 ± 0.44 (9.77 ± 0.44 %ID/g) at 24 h p.i. (*n* = 3, *p* <0.05) with no further change at 48 h p.i. A SUVmean of 1.80 ± 0.69 (5.91 ± 3.31 %ID/g) 24 h p.i. (*n* = 5, *p* <0.01 vs. uptake in OVCAR3 24 h p.i.) was observed in SKOV3 tumors.

In contrast, PET studies using ^64^Cu-labeled anti-CA125 scFv in xenograft mice revealed a SUVmean of 0.64 ± 0.04 (3.21 ± 0.29 %ID/g) 24 h p.i. (*n* = 3) in NIH:OVCAR-3 tumors and 0.25 ± 0.11 (1.49 ± 0.68 %ID/g) (*n* = 3, *p* <0.05) in SKOV3 tumors.

Overall, *in vivo* clearance of the radioimmunoconjugates was evaluated from the heart (representing blood pool content), liver, and kidneys (Additional file 1: Figure S8).

After 24 h, SUVmean in the heart and liver of xenograft animals imaged with ^64^Cu-labeled anti-CA125 MAb was found to be 2.40 0.20 (12.61 ± 1.15 %ID/g) and 2.59 ± 0.20 (12.54 ± 1.67 %ID/g) (*n* = 4), respectively. SUVmean in the kidneys of these animals was 1.64 ± 0.09 (10.35 ± 1.06 %ID/g).

In contrast, injection of ^64^Cu-labeled anti-CA125 scFv led to a SUVmean (*n* = 3) of 0.31 ± 0.06 (1.55 ± 0.40 %ID/g) in the heart, 0.98 ± 0.11 (4.89 ± 0.89 %ID/g) in the liver, and 13.2 ± 1.2 (66.46 ± 9.20 %ID/g) in the kidneys after 24 h. This information demonstrated the different *in vivo* clearance profile between the full-length antibody and the fragment.

### *Ex vivo* analysis

**Figure 7:**
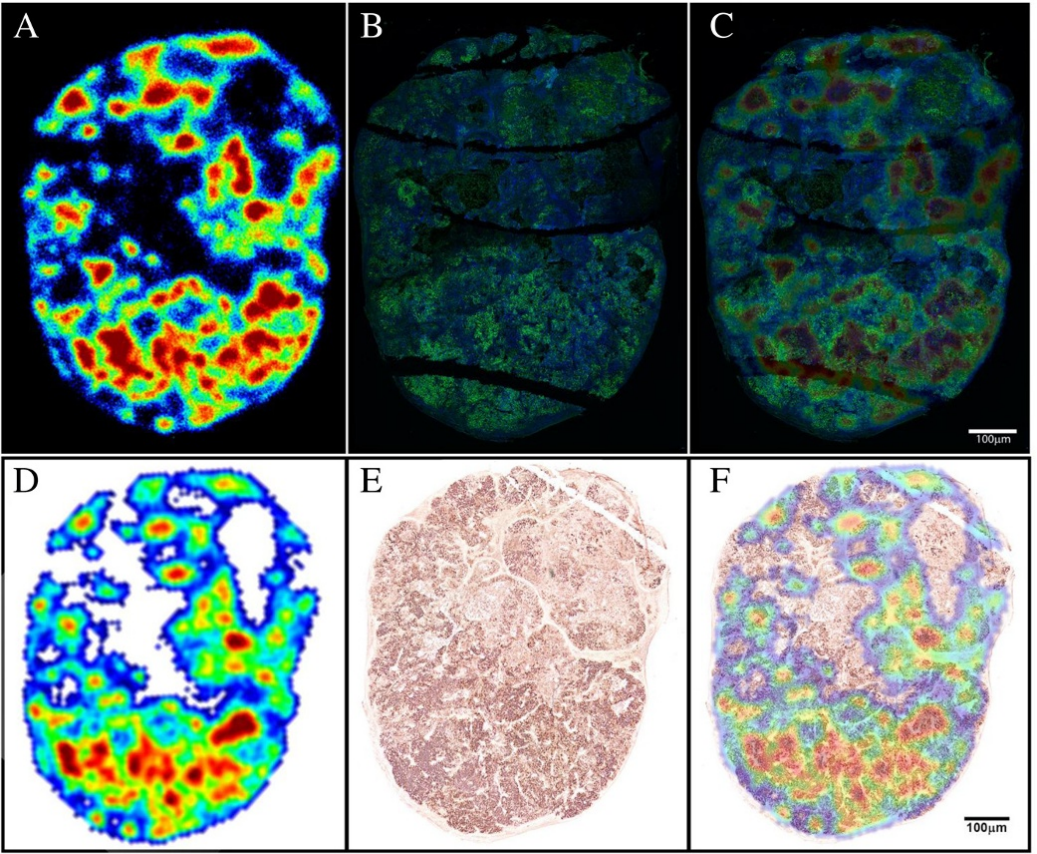
**Autoradiography, immunofluorescence, immunohistochemistry, and overlay images. (A)** Autoradiography image from a section of NIH:OVCAR-3 tumor indicating hot spots of *in vivo* targeting by ^64^Cu-labeled anti-CA125 MAb. **(B)** Immunofluorescence image of the same section staining green in regions of CA125 expression (Alexa Fluor 488) and counterstained blue (Hoechst 33342) for nuclei. **(C)** Overlay of images **(A)** and **(B)** to establish concurrence between signals from independent methods of *ex vivo* analysis to indicate CA125 expression and targeting. **(D)** Autoradiography image from a separate section of NIH:OVCAR-3 tumor indicating hot spots of *in vivo* targeting by ^64^Cu-labeled anti-CA125 MAb. **(E)** Immunohistochemistry image of corresponding tissue section. Intensity of staining is indicative of CA125 expression across the sample. **(F)** Overlay of images **(D)** and **(E)** to establish concurrence between signals from independent methods of *ex vivo* analysis to indicate CA125 expression and targeting.

The NIH:OVCAR-3 tumor tested positive for CA125 expression as seen from immunofluorescence and immunohistochemistry of tissue sections (Figure 7A, B, C, D, E, F).

Immunohistochemistry revealed necrotic and apoptotic regions spanned by connective tissue in the tumor's upper half. Overall, the lower half had more radioactive hot spots, stained stronger for CA125 expression, and had more vasculature surrounded by positively staining tumor foci, which collectively formed pockets of high signals. CA125 staining was observed on the surface of neoplastic cells in the tumor. Hot spots representative of *in vivo* targeting by the radiotracer as seen in autoradiography of tumor slices correlated well with signals from the other two techniques.

A good correlation of all three types of signals was observed in regions of tumor foci interspersed with blood vessels and/or supported by mouse connective tissue.

## Discussion

EOC accounts for more than 90% of all ovarian carcinomas presented in the clinic, and 70% of these cases are high-grade serous ovarian cancers (HGSOC) overexpressing CA125 [25]. This offers a premise to target CA125 for *in vivo* diagnosis of ovarian neoplasms. Here, OVCAR3 human adenocarcinoma cells representative of HGSOCs and SKOV3 cells representative of human clear cell ovarian carcinoma that do not express CA125 were utilized in order to develop a robust preclinical testing platform to validate CA125 expression and targeting. Both the cell lines displayed consistent biochemical characteristics across *in vitro*, *in vivo*, and *ex vivo* analyses.

Unlike a previous report [26], we engineered MAb-B43.13 using a standard bioconjugation strategy to radiolabel it with ^64^Cu towards development of a CA125-targeted radiotracer for immuno-PET. To the best of our knowledge, this is the first systematic report of an immuno-PET strategy for the *in vivo* targeting and imaging of CA125 in EOC. In contrast to OVCAR3 cells in culture, we found the uptake of [^18^ F] FDG in OVCAR3 tumors to be relatively low (Figure 6F), due plausibly to the cystic nature of ovarian tumors *in vivo*[27]. Thus, the utility of a targeted radiotracer that binds to cell surface antigen may be more sensitive and efficient for the *in vivo* delineation of EOC.

In the present work, an isothiocyanate-based bioconjugation of a fluorescent tag and a macrocyclic chelator within prescribed molar ratios to the anti-CA125 MAb and scFv followed by radiolabeling yielded consistent results without compromising their immunoreactivity. This approach offers a scope for labeling MAb-B43.13-based CA125-targeting vectors with different radionuclides, fluorophores, and functional groups for use in diverse imaging and therapeutic applications. Furthermore, based on indications from a previous report [28] and our own observations for non-internalization of surface antigen-bound MAb-B43.13 over 48 h as shown in Figure 3, pre-targeting applications are currently being explored via conjugation of click components to the anti-CA125 MAb-B43.13.

The present study with CA125-targeting MAb-B43.13 and its derivative scFv in preclinical ovarian cancer models found a higher targeted tumor uptake of ^64^Cu-NOTA-MAb-B43.13. Specificity of the observed *in vivo* tumor uptake was validated by pre-dosing OVCAR3 xenograft animals with 1 mg of unmodified anti-CA125 MAb 24 h prior to injection of ^64^Cu-labeled anti-CA125 MAb and scFv. The non-specific tumor uptake observed in our study was attributed to an enhanced permeability and retention effect (EPR) [29], whose numerical value (SUV) fell in range with the uptake of ^64^Cu-NOTA-MAb B43.13 observed in CA125-negative SKOV3 tumors.

The background radiation in all PET imaging studies using ^64^Cu-NOTA-MAb B43.13 could be attributed to a) its longer residency time in circulation, b) consistent non-specific muscle uptake of the radiotracer in all experimental animals (Additional file 1: Figure S9), c) hepatobiliary clearance of the radiotracer and its associated immunocomplexes, and d) potential *in vivo* trans-chelation of ^64^Cu from NOTA by hepatic enzymes such as superoxide dismutase (SOD). However, NOTA has previously demonstrated high *in vivo* kinetic stability as a chelator for ^64^Cu^2+^ ions [30], and our *in vitro* challenge experiments for ^64^Cu-NOTA-MAb B43.13 in the presence of an excess of purified SOD showed negligible trans-chelation (Additional file 1: Figure S10).

In contrast to its favorable *in vitro* targeting properties and proposed advantages, the ^64^Cu-NOTA-scFv-B43.13 was less efficient in its role as a radiotracer for *in vivo* targeting of CA125. These observations are supported by other reports from *in vivo* use of such monomeric antibody fragments [31],[32]. Although the scFv is expected to be renally cleared, we observed maximum renal trapping of the radioimmunoconjugate as early as 1 h p.i. (Additional file 1: Figure S11). Such a first pass effect precludes the scFv's bioavailability for any substantial tumor accumulation at later time points, thus accounting for the lower tumor uptake observed with this vector. This has prompted us to engineer the scFv into a diabody to achieve competitive avidity and better *in vivo* performance.

*Ex vivo* analysis by digital autoradiography reflected successful *in vivo* tumor accumulation of the ^64^Cu-NOTA-MAb-B43.13, while immunohistochemistry provided a panoramic view of CA125 expression in the tumor, indicating regions of high vascularity and stromal components in the tumor architecture. The lower half of the tumor was relatively rich in vasculature and formed suitable sites for neoplastic growth that appeared as densely stained tumor foci. Such vasculature also serves as a route of entry for targeted radioimmunoconjugates into the tumor. Most of the correlating signals from results between the three *ex vivo* techniques described in this study seem localized with strong CA125 staining in areas of dense vascular perfusion. This phenomenon may be a result of the limited penetration of antibody radioimmunoconjugates extravasating across blood vessels that constitute the aberrant tumor vasculature. This is exacerbated by the tumor's interstitial fluid pressure that can further impede the transport of antibodies once they are within the tumor along their pursuit to reach target cells that express CA125 [33]-[35]. Furthermore, the shedding properties of CA125 may create reservoirs of shed antigen within regions of high CA125 expression surrounding the tumor vasculature. This may lead to most of the radiotracer forming high molecular weight immunocomplexes at such junctions that may eventually be cleared via lymphatics.

Although the use of subcutaneous xenograft models of ovarian cancer allowed for easy assessment of tumor burden and an evaluation of the extent of tumor targeting, this advantage also contributes to being a limitation of this study. The next phase of testing this strategy will involve the use of alternative xenograft and/or transgenic animal models that more closely represent human EOC within the natural setting of the peritoneum, accompanied with an inducible recurrence of the disease. However, a drawback to using such a model would be the signal observed from organs of clearance such as the liver and kidneys that may conceal ovarian tumors, closer to these organs in the peritoneal cavity.

A concurrence of *in vitro*, *in vivo*, and *ex vivo* data taken from this preclinical proof-of-concept study represents an initial step towards investigating CA125 as a suitable target for the *in vivo* imaging of EOC. Drawing information from the present study and prior literature reports [12],[36]-[38], it can be speculated that there may be a pathophysiological time interval between the overexpression of CA125 antigen on the surface of ovarian neoplasms and its subsequent shedding into the bloodstream of subjects at high risk for EOC and in recurrence. This time interval could be an existing gap between active small volume disease brewing to relapse out of the peritoneum and the limitations of present-day methods for its detection via CA125 immunoassays. Molecular imaging by immuno-PET may possibly bridge this gap to achieve a timely detection of EOC for better disease management.

## Conclusions

In the work at hand, we have described the synthesis and preclinical radiopharmacological evaluation of ^64^Cu-labeled MAb-B43.13 and its derivative scFv for the PET imaging of CA125 in EOC. This approach offers both an opportunity for early detection of EOC in patients at high risk and a method to monitor patients for recurrence, by providing a non-invasive *in vivo* assessment of CA125 status. A robust model system was developed for preclinical testing, and antibody-based CA125-targeting radioimmunoconjugates were successfully produced with excellent immunoreactivity. Overall, our findings suggest that the more efficient tumor-targeting capabilities and favorable radiopharmacological profiles of the full-length antibody make it a suitable radiotracer for non-invasive *in vivo* evaluation of CA125 in EOC.

## Authors' contributions

SS was responsible for the synthesis and (radio) pharmacological evaluation of the immunoconjugates. He was responsible for writing the manuscript. MeW was responsible for animal experiments, including analysis of the PET data. MoW was supporting all radiopharmacological experiments. DG supported *ex vivo* analyses and immunofluorescence and immunohistochemistry experiments from tumor sections. BA supported cell culture of OVCAR3 and SKOV3 cells. SEL supported radiolabeling experiments. FW was responsible for the design of the study and critically revised the manuscript. All authors read and approved the final manuscript.

## Additional file

## Electronic supplementary material

Additional file 1:**Supporting information.** A file containing supplementary table and figures. (DOCX 3 MB)

Below are the links to the authors’ original submitted files for images.

Authors’ original file for figure 1

Authors’ original file for figure 2

Authors’ original file for figure 3

Authors’ original file for figure 4

Authors’ original file for figure 5

Authors’ original file for figure 6

Authors’ original file for figure 7

## Abbreviations

%ID/g: percentage of injected dose per gram tissue
CA125: cancer antigen 125
EOC: epithelial ovarian cancer
MAP: maximum *a priori*
PET: positron emission tomography
ROI: region of interest, reversed phase
SEM: standard error of means
SUV: standardized uptake value
TAC: time-activity curve
