# Supplementary material for: Immuno-PET of epithelial ovarian cancer: harnessing the potential of CA125 for non-invasive imaging
Source: EJNMMI Res. 2014 Nov 12;4:60. doi: 10.1186/s13550-014-0060-4 (PMC4883985; doi:10.1186/s13550-014-0060-4)
Supplement: Supplementary file 1 — Additional file 1:Supporting information. A file containing supplementary table and figures. (DOCX 3 MB) [file 13550_2014_60_MOESM1_ESM.docx]

**Supporting Information**

**Immuno-PET of Epithelial Ovarian Cancer: Harnessing the potential of CA125 for non-invasive imaging**

*Sai Kiran Sharma, Melinda Wuest, Monica Wang, Darryl Glubrecht, Bonnie Andrais, Suzanne E Lapi, Frank R Wuest*


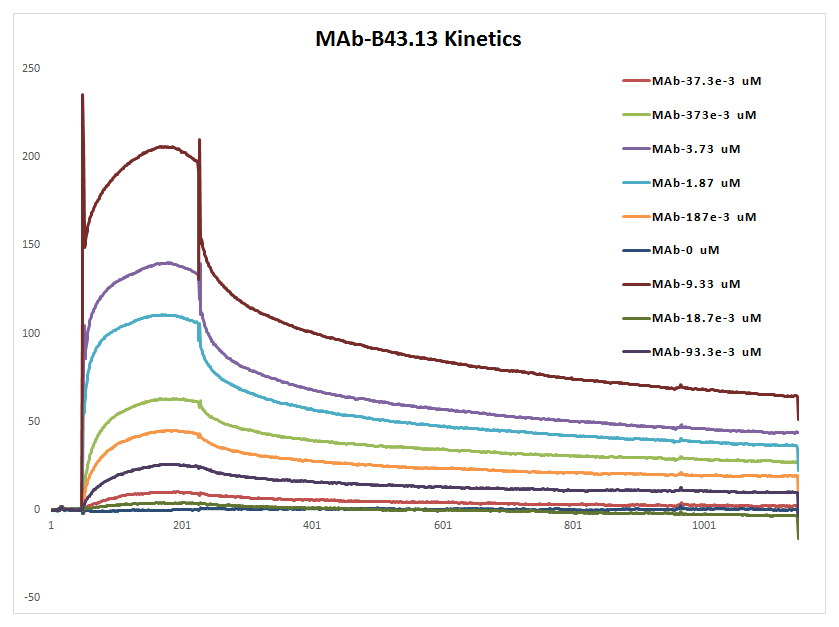
**Surface Plasmon Resonance**

Sensorgrams of the raw kinetic data for the various concentrations of MAb-B43.13 tested for binding to CA125 on a CM5 chip are represented in the following figure. The concentrations tested are listed in the legend of the graph:

**Figure S1.1**


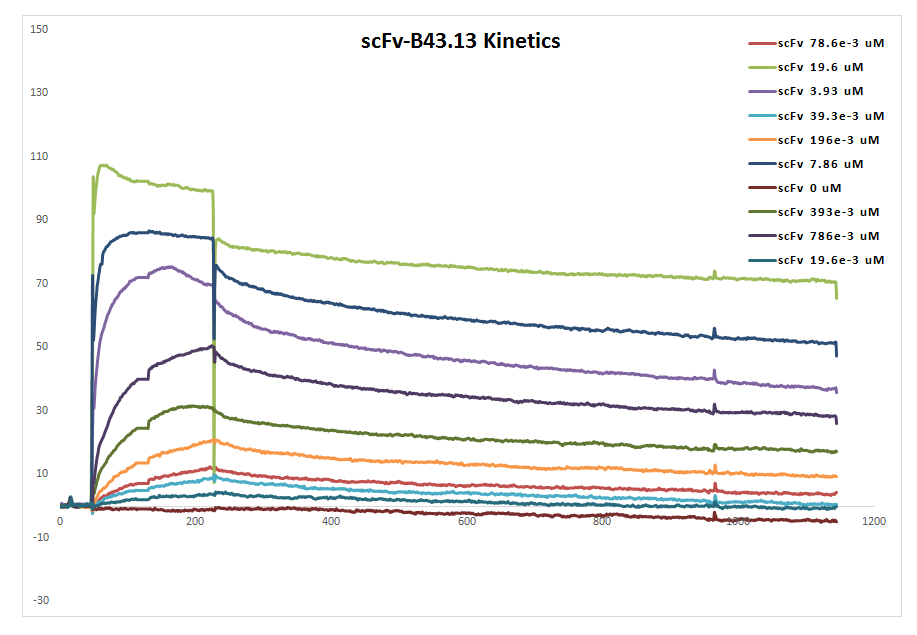
Sensorgrams of the raw kinetic data for the various concentrations of scFv-B43.13 tested for binding to CA125 on a CM5 chip are represented in the following figure. The concentrations tested are listed in the legend of the graph:

**Figure S1.2**

**Table S1: Binding Affinity Constants**

| **Analyte** | **k_a_ (M^-1^ s-^1^)** | **k_d (_s^-1^)** | **K_A_ (M^-1^)** | **K_D_ (M)** |
| --- | --- | --- | --- | --- |
| **MAb-B43.13** | **3.41 x 10^5^** | **8.73 x 10^-4^** | **3.9 x 10^8^** | **2.56 x 10^-9^** |
| **scFv-B43.13** | **4.77 x 10^4^** | **5.28 x 10^-4^** | **9.03 x 10^7^** | **1.11 x 10^-8^** |

**Determination of Bi-functional Chelates per MAb/scFv**

1. NOTA conjugated anti-CA125 MAb and scFv were analyzed by MALDI-ToF-MS/MS at the Institute for Biomolecular Design, University of Alberta, to determine the number of bi-functional chelate molecules per targeting vector. 1 µL of each sample was mixed with 1 µL of sinapic acid (10 mg/ml in 50% acetonitrile:water and 0.1% trifluoroacetic acid). 1 µL of the sample/matrix solution was then spotted onto a stainless steel target plate and allowed to air dry. All mass spectra were obtained using a Bruker Ultraflex MALDI-ToF/ToF (Bruker Daltonic GmbH). Ions were analyzed in positive mode and external calibration was performed by use of a standard protein mixture.


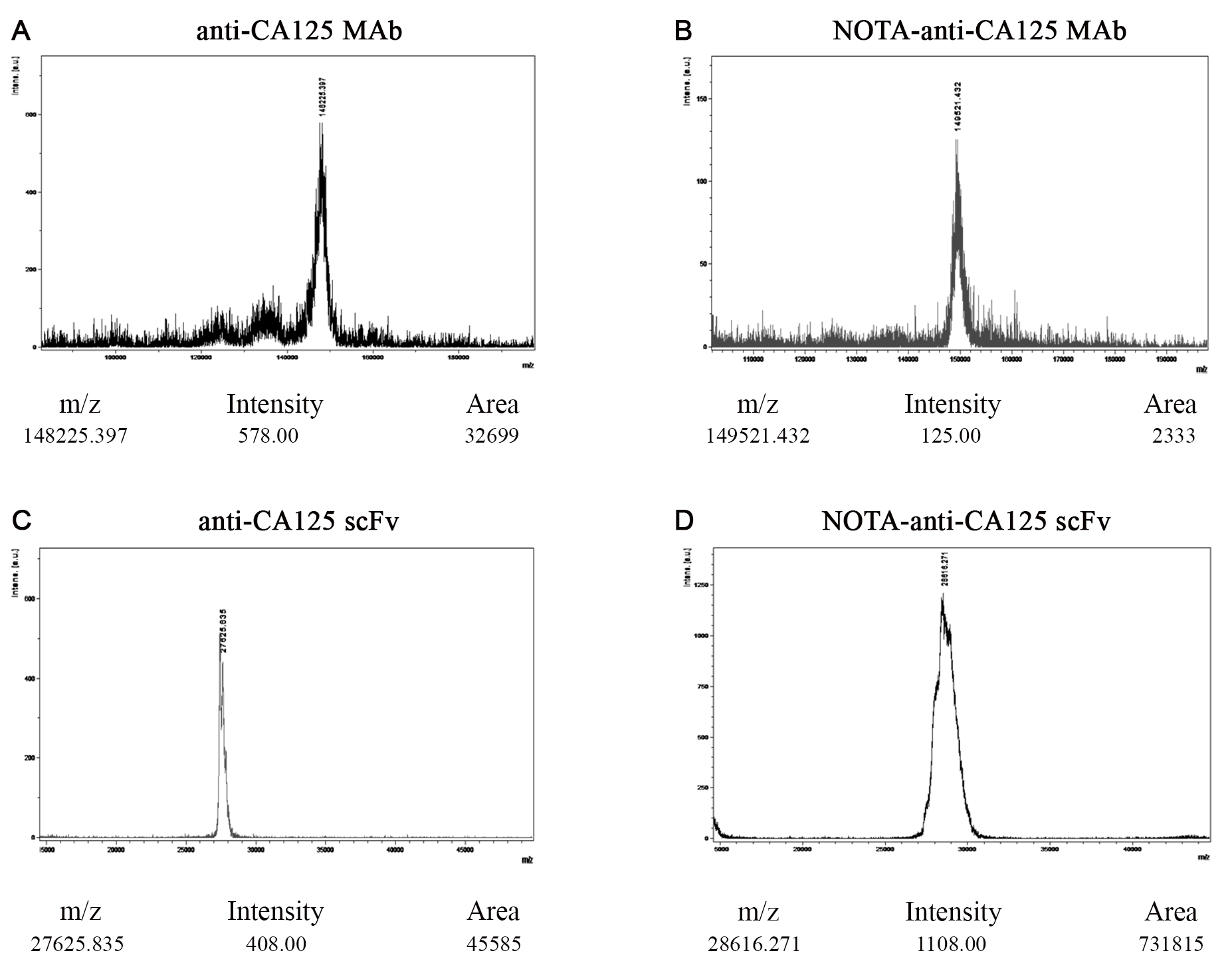


**Figure S2**: Chromatograms from MALDI-ToF analysis of **A)** anti-CA125 MAb; **B)** NOTA-conjugated anti-CA125 MAb; **C)** anti-CA125 scFv; **D)** NOTA-conjugated anti-CA125 scFv. The number of NOTA molecules conjugated per MAb/scFv was calculated by dividing the difference between the masses of the NOTA-conjugated versions and un-conjugated counterparts by the molecular weight of NOTA (559.9 Da).

1. An isotopic dilution assay as per the method of Cooper et al (1) was also performed to verify data obtained from MALDI-ToF MS/MS analysis of immunoconjugates. Percentage radiolabeling efficiency was plotted as a function of different moles of copper added to a fixed concentration (moles) of MAb/scFv to determine the average number of bi-functional chelates conjugated per targeting vector.


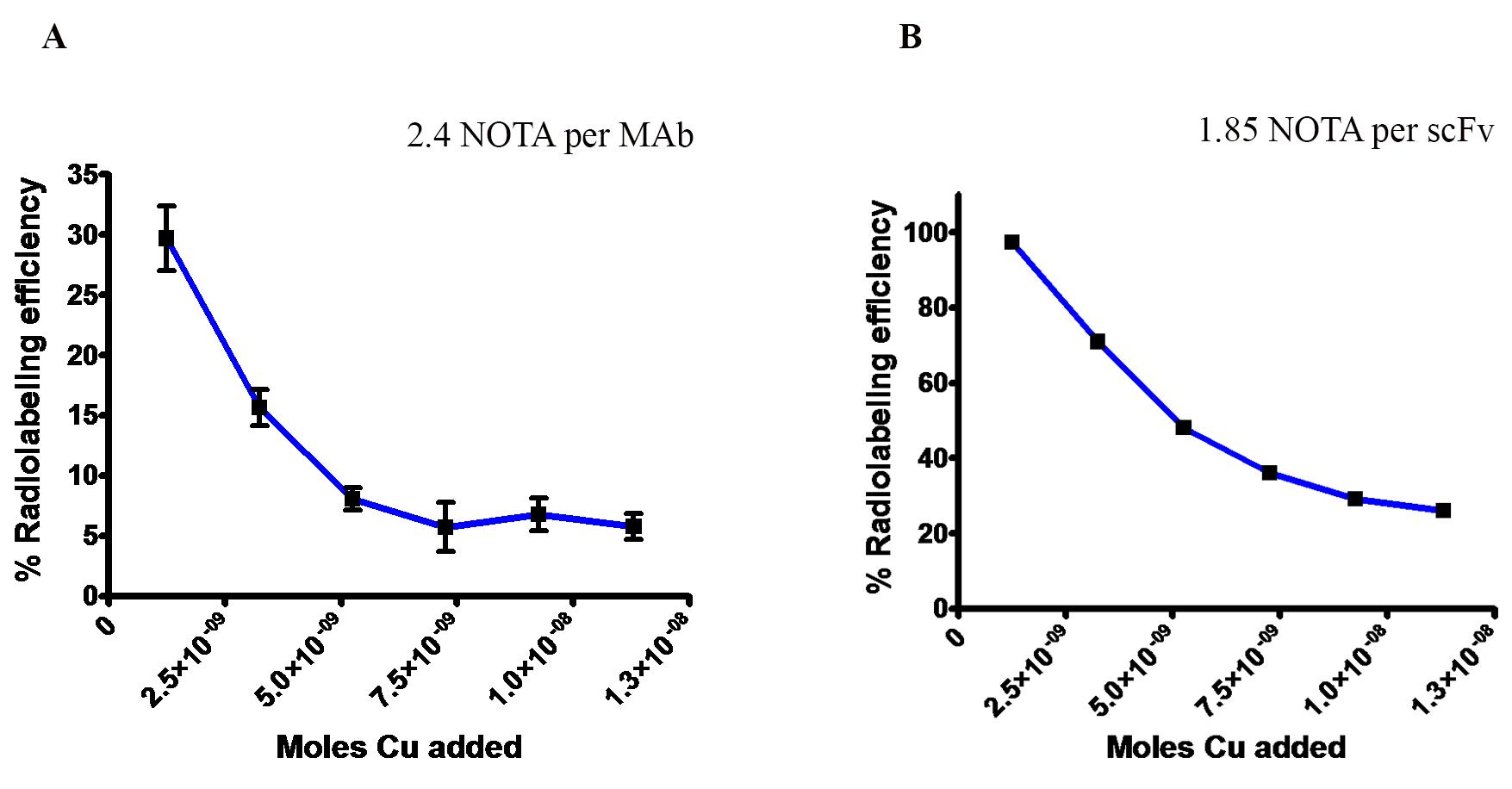


**Figure S3**: The number of bi-functional chelators (BFCs) per **A)** MAb-B43.13 and **B)** scFv-B43.13 was determined by plotting the radiolabeling efficiencies obtained using different concentrations of ^64^Cu and immunoconjugate. Knowing the number of moles of ^64^Cu added initially, the % radiolabeling efficiency was used to indirectly derive the number of moles of ^64^Cu chelated to the immunoconjugate. The moles of ^64^Cu chelated are divided by the (fixed) moles of MAb-B43.13 (0.25 nmols) or scFv-B43.13 (1.285 nmols) added per radiolabeling reaction. Finally, the average ratio across different concentrations is considered as the number of BFCs conjugated per MAb-B43.13 or scFv-B43.13 molecule.

**Quality control of purified radioimmunoconjugates**

^64^Cu-labeled anti-CA125 MAb and scFv were purified by size exclusion chromatography using a pre-packed desalting column. The radioimmunoconjugates eluted out in the earlier fractions owing to their molecular size compared to EDTA chelated ^64^Cu from the reaction mixture. 1 µL of the quenched radiolabeling reaction mixtures and selected fractions from size exclusion purification were spotted on ITLC-SG strips, allowed to dry and separate using 10mM EDTA pH 5.5 as the eluent before analysis on a radio-TLC scanner (Bioscan AR-2000). As per previously established results in such experiments for quality control, ^64^Cu-labeled anti-CA125 MAb and scFv remained at the baseline and any un-conjugated or EDTA-chelated ^64^Cu moved with the solvent front on ITLC strips.

Additionally, 15 μl of each elution fraction was electrophoresed on a 12% SDS-PAGE gel under non-reducing conditions and evaluated by autoradiography on a BAS-5000 phoshorimager (Fujifilm).


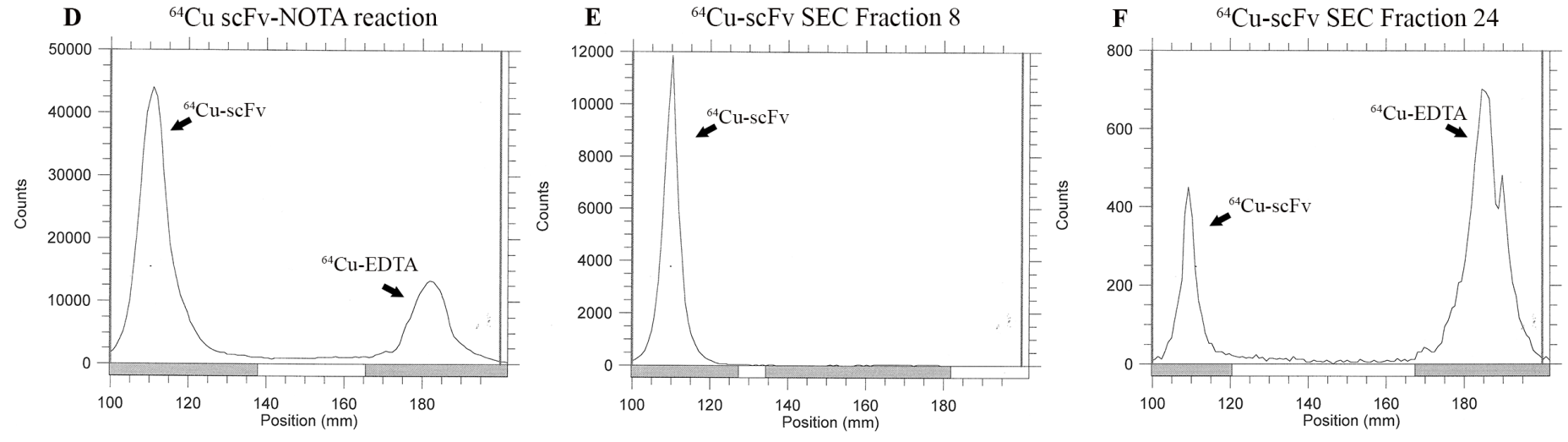

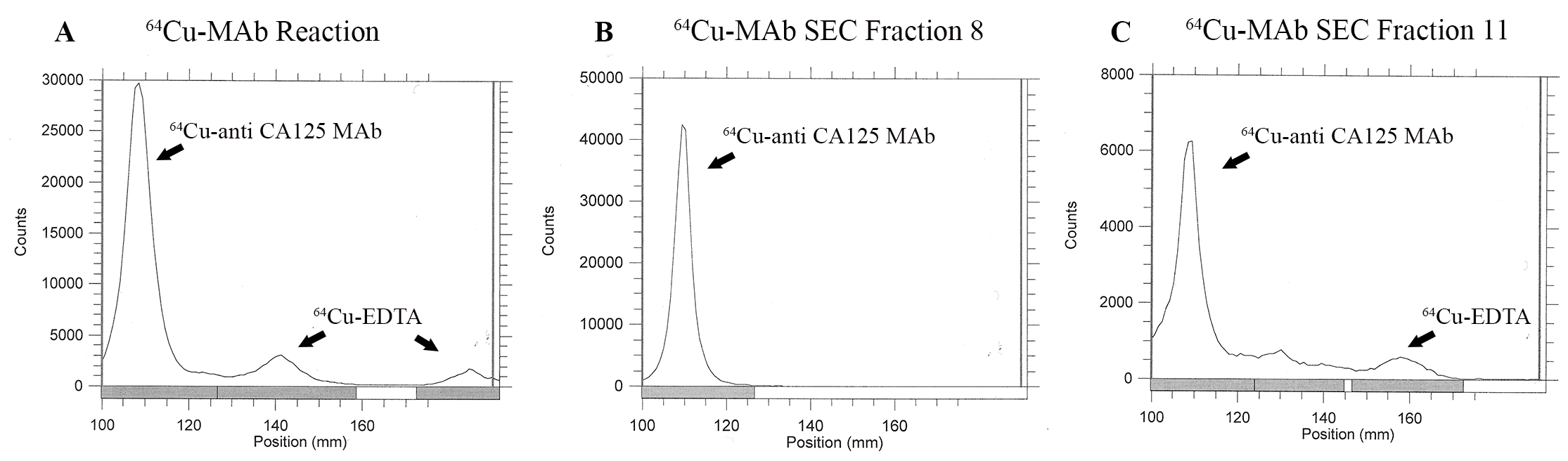


**Figure S4:** Radio-TLCs of **A)** crude reaction mixture from ^64^Cu-labeling of NOTA-MAb-B43.13; **B)** Fraction 8 from SEC-purified ^64^Cu-NOTA-MAb-B43.13; **C)** Fraction 11 from SEC-purified ^64^Cu-NOTA-MAb-B43.13; **D)** crude reaction mixture from ^64^Cu-labeling of NOTA-scFv-B43.13; **E)** Fraction 8 from SEC-purified ^64^Cu-NOTA-scFv-B43.13; **F)** Fraction 24 from SEC-purified ^64^Cu-NOTA-scFv-B43.13. The antibody-based radioimmunoconjugate construct remains at the baseline (extreme left of the TLC plate), while ^64^Cu ions and [^64^Cu]-EDTA elute with the solvent front (far right side of each TLC plate).


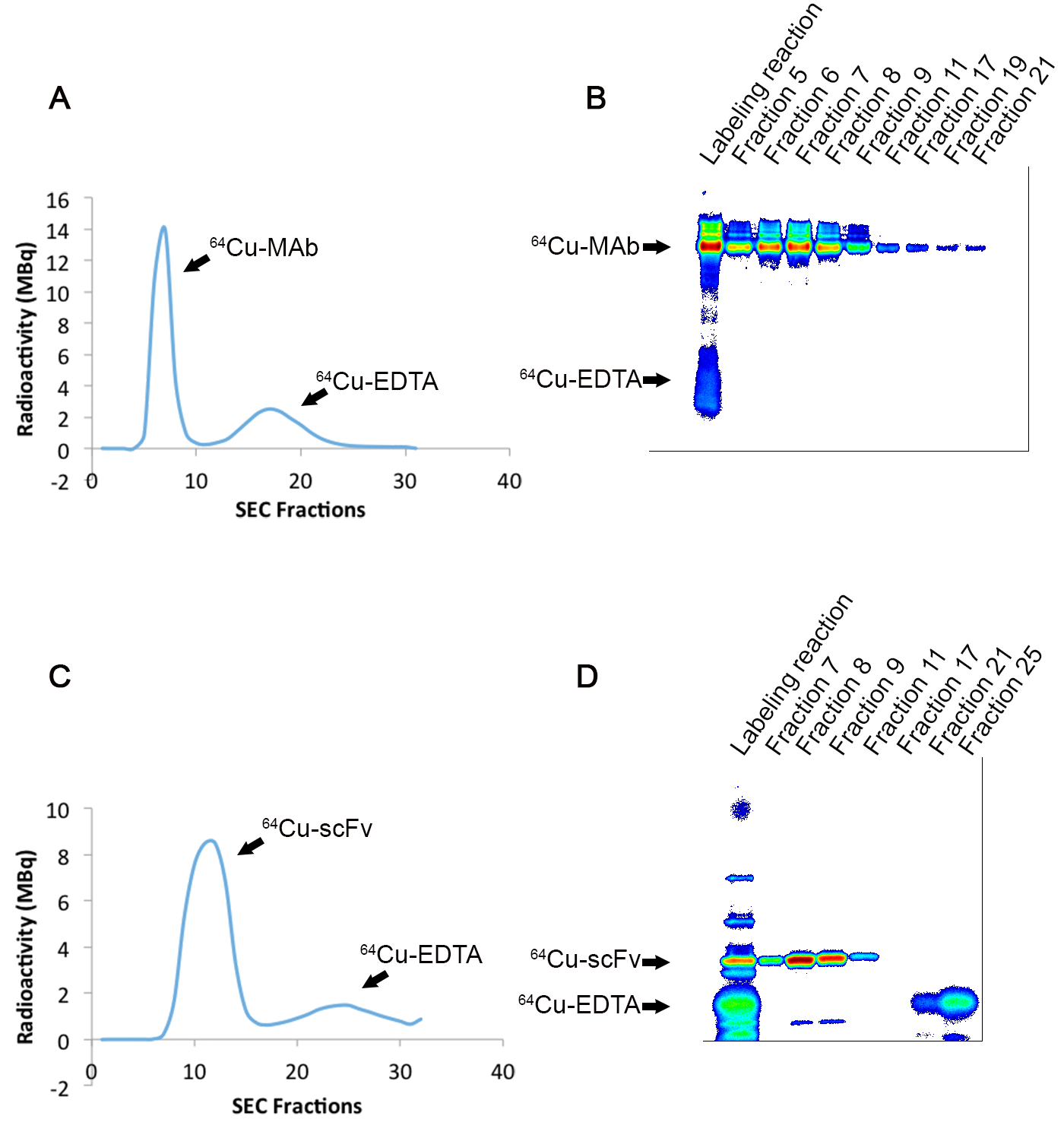


**Figure S5**: **A)** Size exclusion purification histograms of ^64^Cu-labeled anti-CA125 MAb. **B)** Phosphorimage of purified ^64^Cu-labeled anti-CA125 MAb fractions upon SDS-PAGE**. C)** Size exclusion purification histograms of ^64^Cu-labeled anti-CA125 scFv. **D)** Phosphorimage of purified ^64^Cu-labeled anti-CA125 scFv fractions upon SDS-PAGE**.**

**Stability** **of** **radioimmunoconjugates**

^64^Cu-labeled MAb and scFv was analyzed by incubating 5 MBq of purified radio-immunoconjugates with 500 µl human AB type serum at 37°C for 48 h. Samples were analyzed by size exclusion HPLC at 0, 24 and 48 h on a Biosep SEC-S-2000 column (Phenomenex) using an isocratic mobile phase of 0.1 M phosphate buffer at a flow rate of 1 ml/min and by ITLC using 10 mM EDTA pH 5.5 as the eluent or via ITLC.


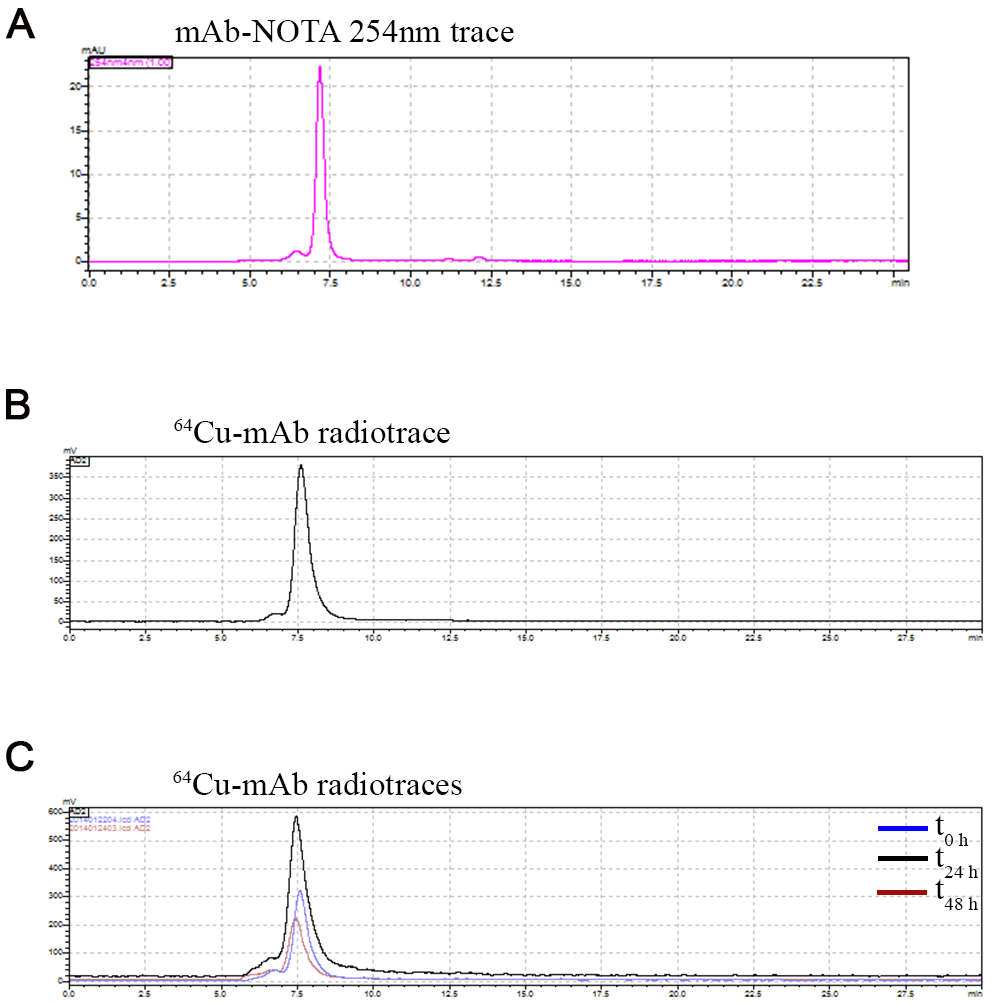


**Figure S6**: **A)** Analytical HPLC UV-trace of NOTA-MAb-B43.13; **B)** Radio-HPLC trace of ^64^Cu-NOTA-MAb-B43.13 at t = 0 h; **C)** Overlay of radio-HPLC traces of ^64^Cu-NOTA-MAb-B43.13 at t=0 h, 24 h and 48 h to analyze any degradation or loss of ^64^Cu from the radioimmunoconjugate upon incubation in serum for the indicated periods of analysis.


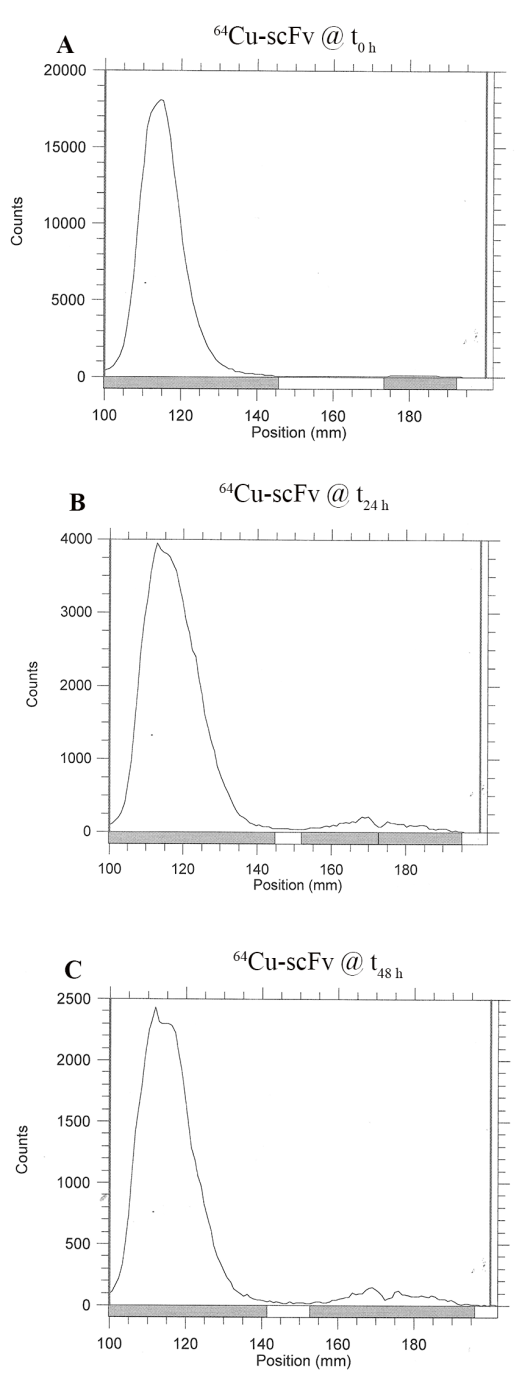


**Figure S7**: Radio-TLCs of ^64^Cu-NOTA-scFv-B43.13 at **A)** t = 0 h; **B)** t = 24 h; **C)** t = 48 h.

***In vivo* Clearance profiles of radioimmunoconjugates**:

**
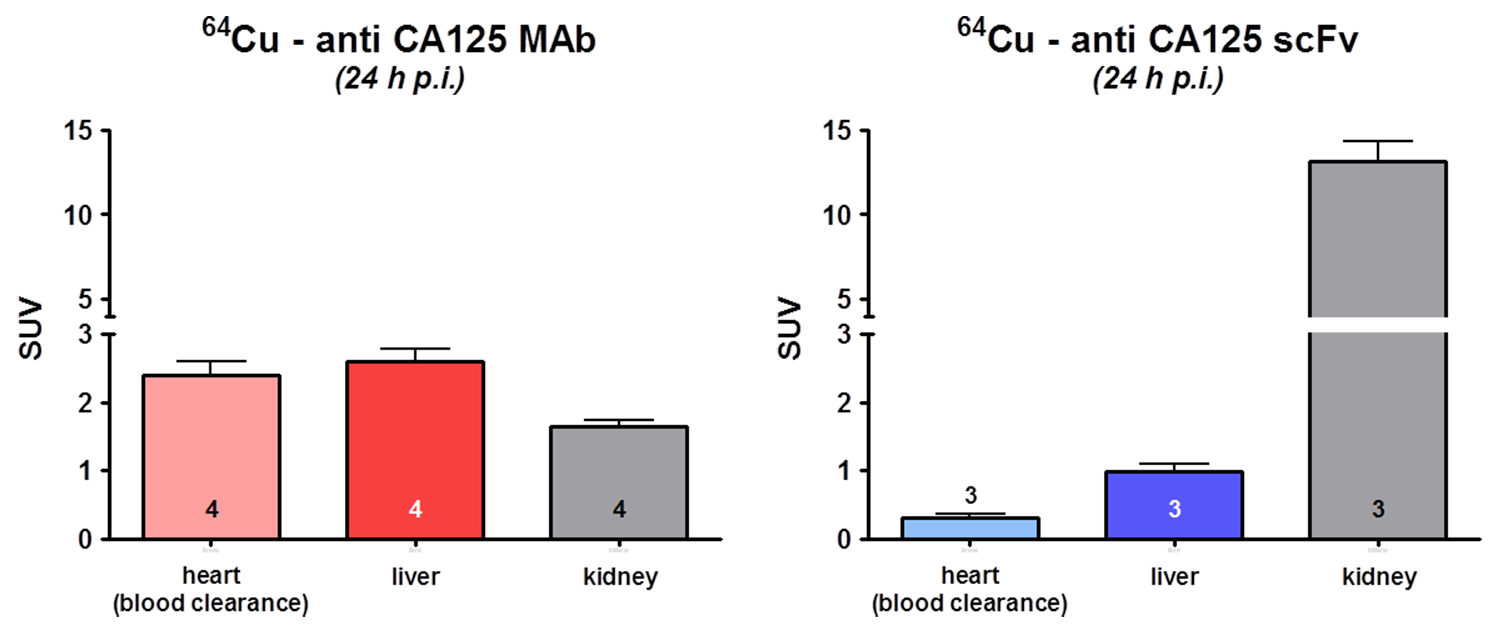
**

**Figure S8**: Bar graphs representing the different *in vivo* biodistribution and clearance profiles between ^64^Cu-labeled anti-CA125 MAb vs. scFv in OVCAR3 xenograft mice. Number of animals (n) used per analysis is indicated at the bottom of each bar.

**Muscle Uptake and Tumor-to-Muscle Ratios**

Muscle SUV*_mean_* at 24 h p.i were comparable between injections of ^64^Cu-labeled anti-CA125 MAb (0.46 ± 0.07; n = 4), in blocked experiments (0.45 ± 0.05; n = 4) and non-specific ^64^Cu-isotype IgG (0.44 ± 0.04; n = 3), respectively. Tumor-to-muscle ratios calculated at 24 h p.i for ^64^Cu-labeled anti-CA125 MAb was found to be 13.07 ± 1.72 (n = 4); 6.85 ± 1.73 (n = 4) in animals used for blocking experiments and 4.12 ± 0.49 (n = 3) in animals injected with isotype radioimmunoconjugate. Tumor-to-muscle ratios calculated at 24 h p.i for ^64^Cu-labeled anti-CA125 scFv were found to be 5.03 ± 0.77 (n = 3) and 4.41 (n = 1) in the test and blocking experiments respectively.

**
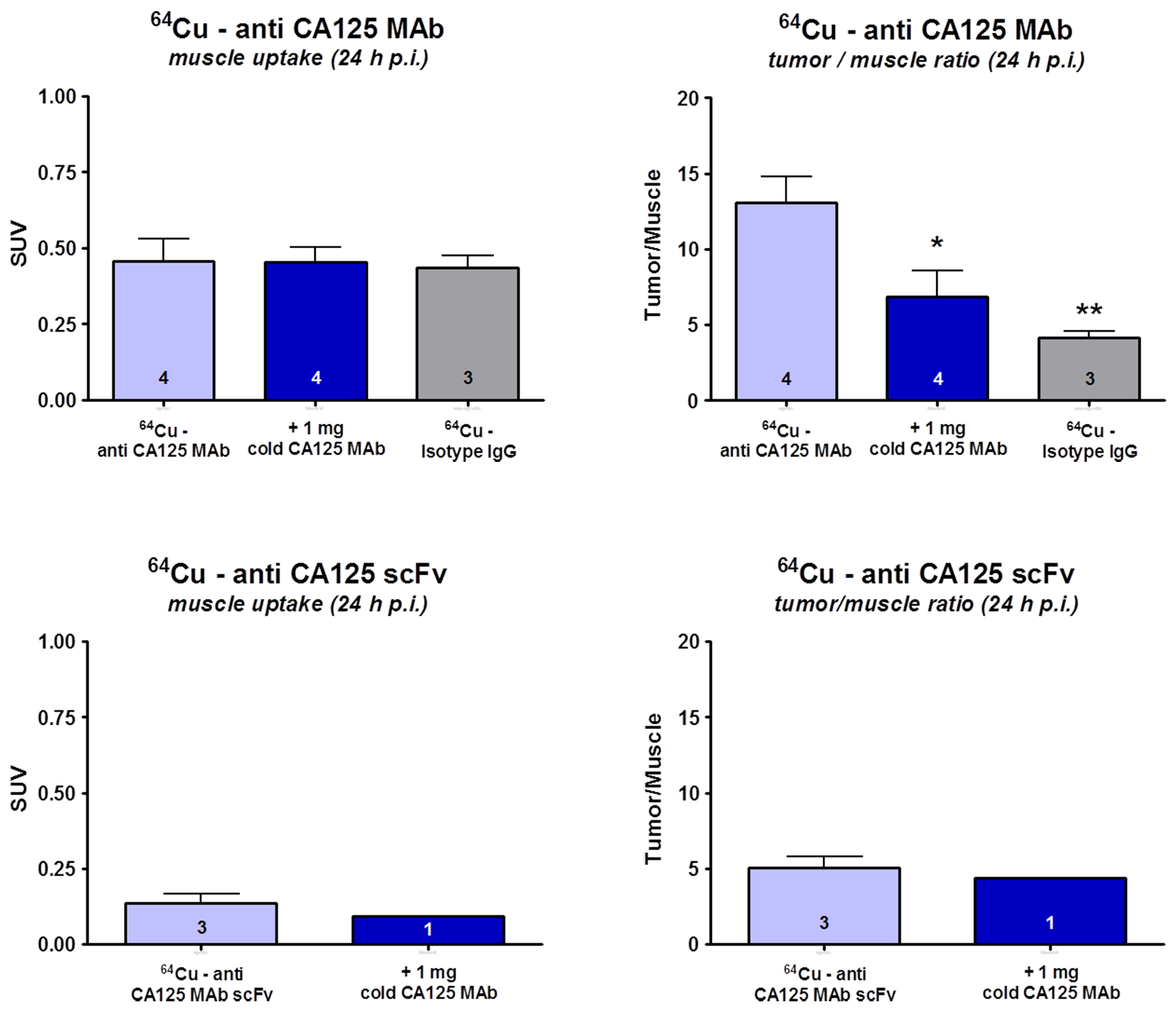
**

**Figure S9**: Bar graphs representing the muscle uptake of ^64^Cu-labeled anti-CA125 MAb and scFv and their corresponding tumor-to-muscle ratios in OVCAR3 xenograft mice. Data deduced from immuno-PET images. Number of animals (n) per analysis is indicated at the bottom of each bar. * p < 0.05, ** p< 0.01.

**SOD Challenge experiments**

^64^Cu-labeled anti-CA125 MAb (5MBq) was challenged with a 1000 fold excess of Superoxide dismutase (Sigma) and allowed to incubate at 37°C over a period of 48 h. Samples were analyzed for trans-chelation by size exclusion chromatography using an SEC-2000 on a Shimadzu analytical HPLC using conditions mentioned above.

**
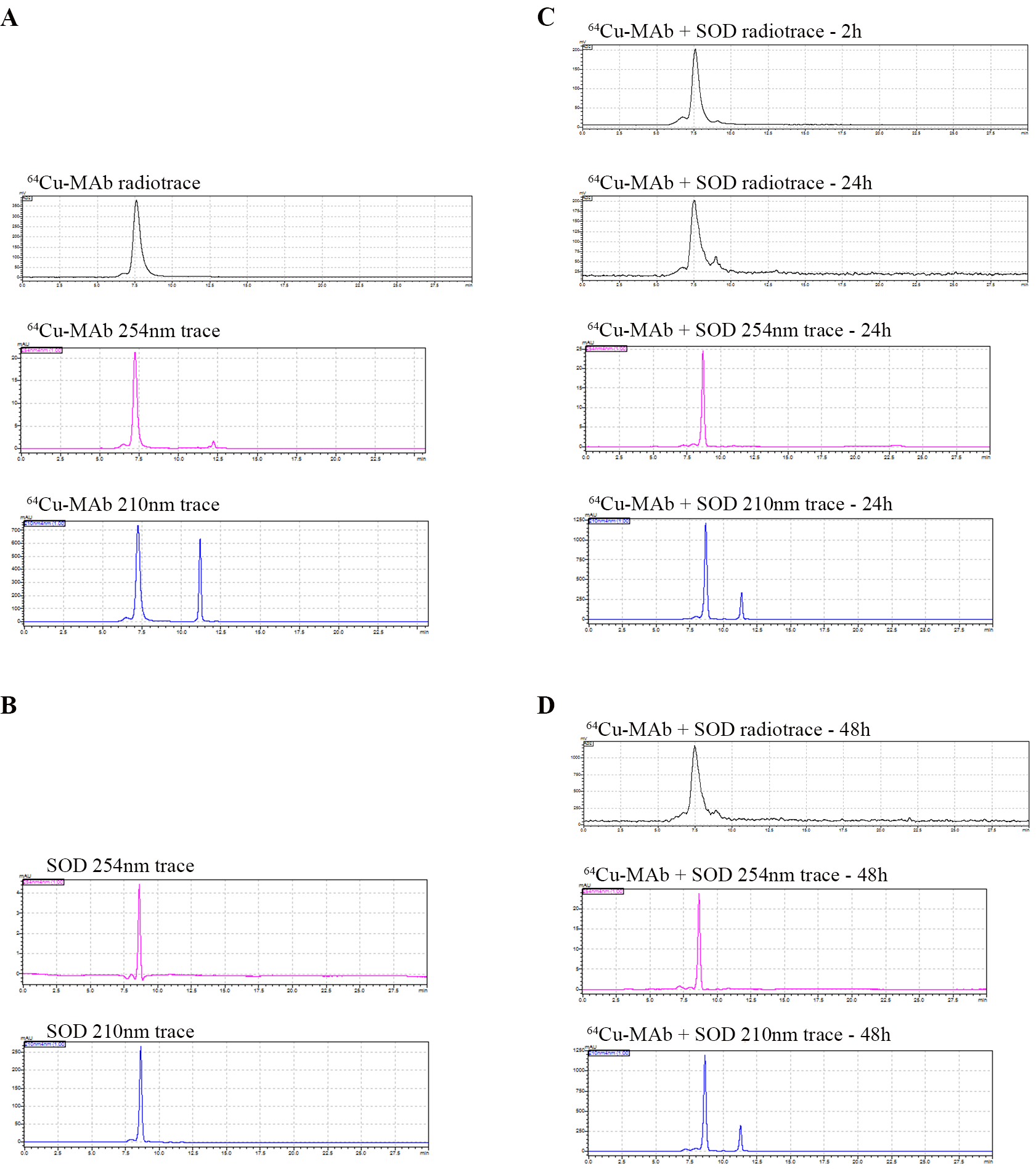
**

**Figure S10**: Analytical Radio and UV-traces from size exclusion HPLC showing elution profile and retention times for **A)** ^64^Cu-NOTA-MAb-B43.13; **B)** UV-traces for Superoxide Dismutase (SOD); **C)** ^64^Cu-NOTA-MAb-B43.13 challenged with excess SOD to evaluate trans-chelation of ^64^Cu after incubation in 0.25M sodium acetate for 24 h; **D)** ^64^Cu-NOTA-MAb-B43.13 challenged with excess SOD to evaluate trans-chelation of ^64^Cu after incubation in 0.25M sodium acetate for 24 h.

**^64^Cu-scFv PET imaging**:

Small animal PET imaging of OVCAR3 xenograft mice with ^64^Cu-labeled scFv revealed renal trapping as early as 1 h p.i..


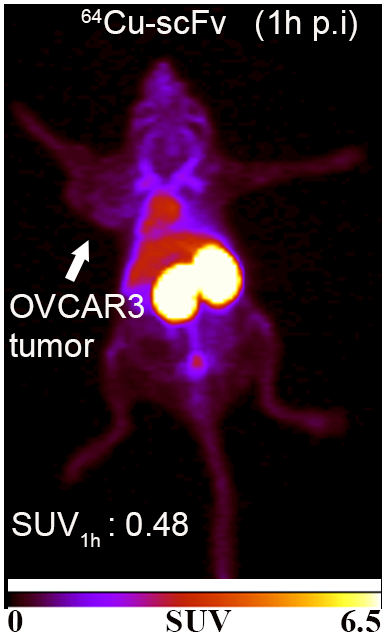


**Figure S11**: Small animal PET image of an OVCAR3 xenografted BALB/c nude mouse taken 1 h post-injection with 6 MBq of ^64^Cu-NOTA-scFv-B43.13.

**References**:

1. Cooper MS, et al. (2012) Comparison of ^64^Cu-complexing bifunctional chelators for radioimmunoconjugation: labeling efficiency, specific activity, and in vitro/in vivo stability. *Bioconjug Chem* 23(5):1029-39.
