# Supplementary figures and images for: Immuno-PET of epithelial ovarian cancer: harnessing the potential of CA125 for non-invasive imaging
Source: EJNMMI Res. 2014 Nov 12;4:60. doi: 10.1186/s13550-014-0060-4 (PMC4883985; doi:10.1186/s13550-014-0060-4)

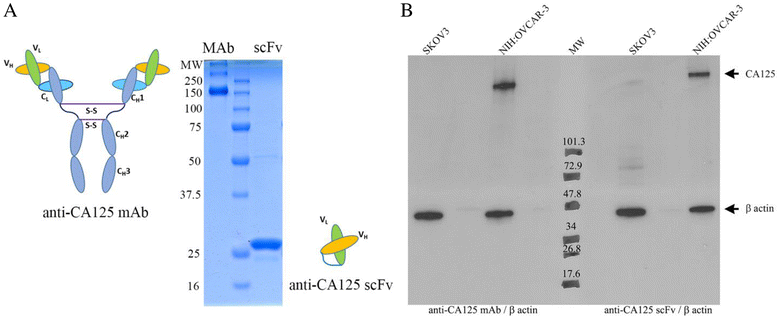

Supplement: Supplementary file 2 — Authors’ original file for figure 1 [file 13550_2014_60_MOESM2_ESM.gif]

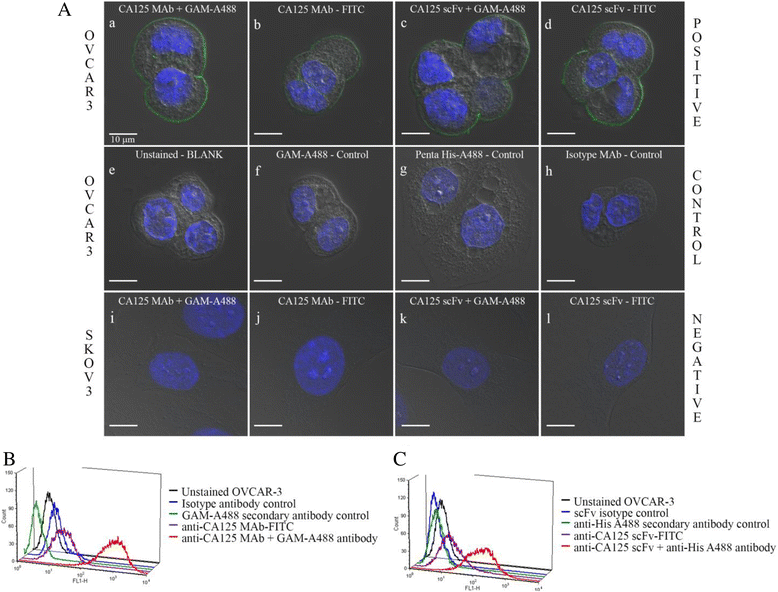

Supplement: Supplementary file 3 — Authors’ original file for figure 2 [file 13550_2014_60_MOESM3_ESM.gif]

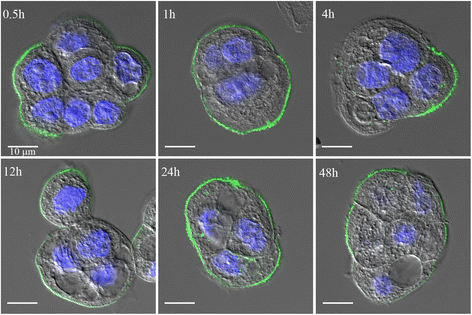

Supplement: Supplementary file 4 — Authors’ original file for figure 3 [file 13550_2014_60_MOESM4_ESM.gif]

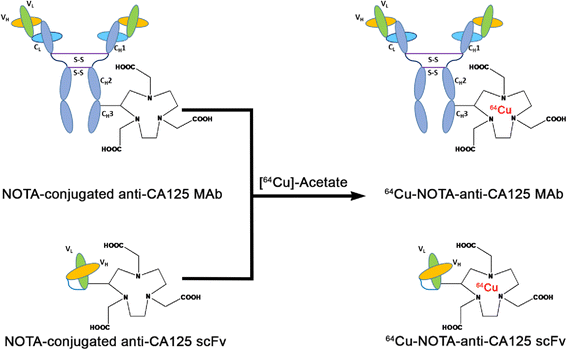

Supplement: Supplementary file 5 — Authors’ original file for figure 4 [file 13550_2014_60_MOESM5_ESM.gif]

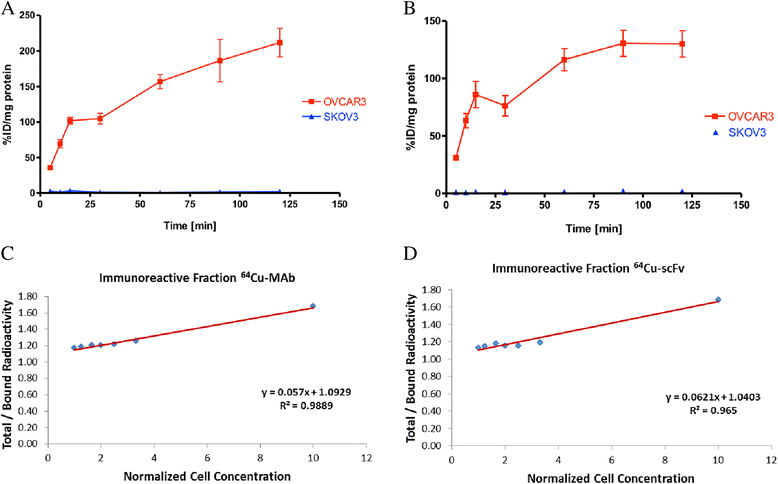

Supplement: Supplementary file 6 — Authors’ original file for figure 5 [file 13550_2014_60_MOESM6_ESM.gif]

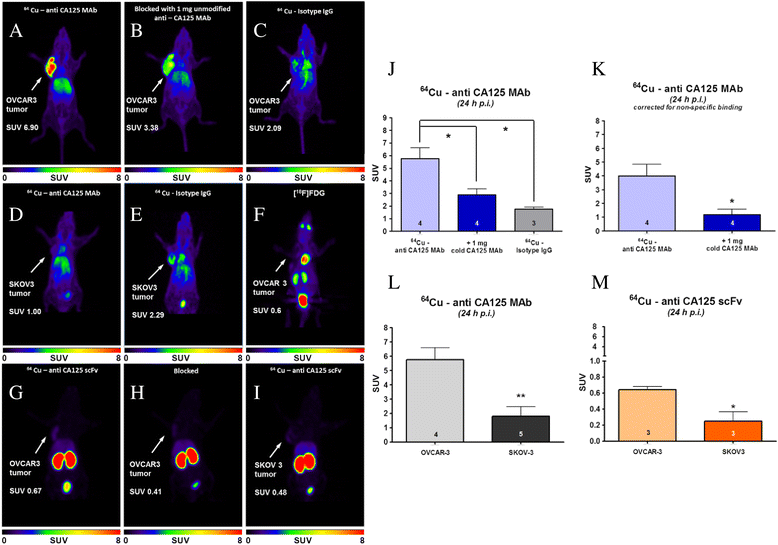

Supplement: Supplementary file 7 — Authors’ original file for figure 6 [file 13550_2014_60_MOESM7_ESM.gif]

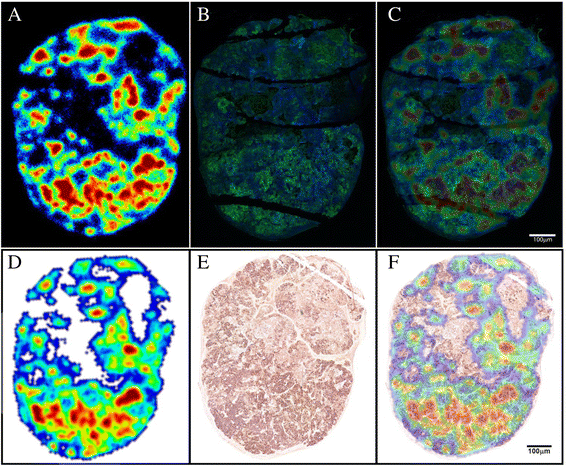

Supplement: Supplementary file 8 — Authors’ original file for figure 7 [file 13550_2014_60_MOESM8_ESM.gif]
